# Tricyclic Nucleobase Analogs and Their Ribosides as Substrates and Inhibitors of Purine-Nucleoside Phosphorylases III. Aminopurine Derivatives

**DOI:** 10.3390/molecules25030681

**Published:** 2020-02-05

**Authors:** Alicja Stachelska-Wierzchowska, Jacek Wierzchowski, Michał Górka, Agnieszka Bzowska, Ryszard Stolarski, Beata Wielgus-Kutrowska

**Affiliations:** 1 Department of Physics and Biophysics, University of Warmia and Mazury in Olsztyn, 10-719 Olsztyn, Poland; jacek.wie@uwm.edu.pl; 2 Division of Biophysics, Institute of Experimental Physics, Faculty of Physics, University of Warsaw, 5 Pasteura St., 02-093 Warsaw, Poland; michal.gorka@fuw.edu.pl (M.G.); Agnieszka.Bzowska@fuw.edu.pl (A.B.); Ryszard.Stolarski@fuw.edu.pl (R.S.); 3 Biological and Chemical Research Centre, University of Warsaw, 101 Zwirki i Wigury St., 02-089 Warsaw, Poland

**Keywords:** purine nucleoside phosphorylase, nucleobase/nucleoside analogs, chemo-enzymatic synthesis, fluorescence, NMR, enzyme-substrate complexes

## Abstract

Etheno-derivatives of 2-aminopurine, 2-aminopurine riboside, and 7-deazaadenosine (tubercidine) were prepared and purified using standard methods. 2-Aminopurine reacted with aqueous chloroacetaldehyde to give two products, both exhibiting substrate activity towards bacterial (*E. coli*) purine-nucleoside phosphorylase (PNP) in the reverse (synthetic) pathway. The major product of the chemical synthesis, identified as 1,N^2^-etheno-2-aminopurine, reacted slowly, while the second, minor, but highly fluorescent product, reacted rapidly. NMR analysis allowed identification of the minor product as N^2^,3-etheno-2-aminopurine, and its ribosylation product as N^2^,3-etheno-2-aminopurine-N^2^-β-d-riboside. Ribosylation of 1,N^2^-etheno-2-aminopurine led to analogous N^2^-β-d-riboside of this base. Both enzymatically produced ribosides were readily phosphorolysed by bacterial PNP to the respective bases. The reaction of 2-aminopurine-N^9^-β -d-riboside with chloroacetaldehyde gave one major product, clearly distinct from that obtained from the enzymatic synthesis, which was not a substrate for PNP. A tri-cyclic 7-deazaadenosine (tubercidine) derivative was prepared in an analogous way and shown to be an effective inhibitor of the *E. coli*, but not of the mammalian enzyme. Fluorescent complexes of amino-purine analogs with *E. coli* PNP were observed.

## 1. Introduction

Tricyclic analogs of natural purine bases and their glycosides have been applied as fluorescent probes in the investigations of structure and function of nucleic acids (DNA, RNA) and enzymes related to nucleic acid metabolism and/or those utilizing nucleotide cofactors [1,2,3,4,5,6]. The most popular probe of this kind is 1,N^6^-etheno-adenosine (εAdo), prepared from adenosine reacting with chloroacetaldehyde (CAA), and related nucleotides [1]. Tri-cyclic nucleosides and nucleotides are also utilized as dimensional probes for enzymatic studies [5,6]. Some of the tri-cyclic analogs and their derivatives reveal promising anti-viral properties [7], recently reviewed by Janz-Wechmann et al. [8,9]. They are known to reveal substrate or inhibitory activities towards many enzymes of purine metabolism [2], and they are important intermediates in the process of chemical mutagenesis [10,11].

Purine-nucleoside phosphorylase (PNP, E.C. 2.4.2.1) catalyzes a reversible phosphorolysis of many nucleosides to the respective bases [12]. It belongs to a group of enzymes involved in the purine salvage pathway, present in the majority of higher organisms [12], which is the only source of these indispensable building blocks of DNA and RNA for microorganisms lacking *de novo* purine nucleoside synthesis. PNP is responsible for the regulation of the nucleoside concentrations within the living cells, and it is a target of various types of pharmaceutical interventions [12,13], including gene therapy of some inherited immunological deficiencies [14] and gene therapy of solid tumors [15]. Additionally, PNP’s from various sources are utilized as biocatalysts in chemo-enzymatic syntheses of various nucleoside analogs of pharmaceutical and/or analytical significance [16,17,18,19,20]. Our previous investigations have shown that PNP isolated from *E. coli*, which is known to possess a broad specificity toward various base and nucleoside analogs [16], is also active toward many tri-cyclic nitrogen bases, derived from adenine and guanine [17,21,22], producing in the phosphate free media many potentially useful ribosides, using β-d-ribose-1-phosphate (R1P) as a ribose source. The reverse, phosphorolytic reactions are, in some cases, as rapid as the analogous reactions with natural substrates, like adenosine or guanosine [21,22], and therefore can possess analytical applications. 

**Figure 1:**
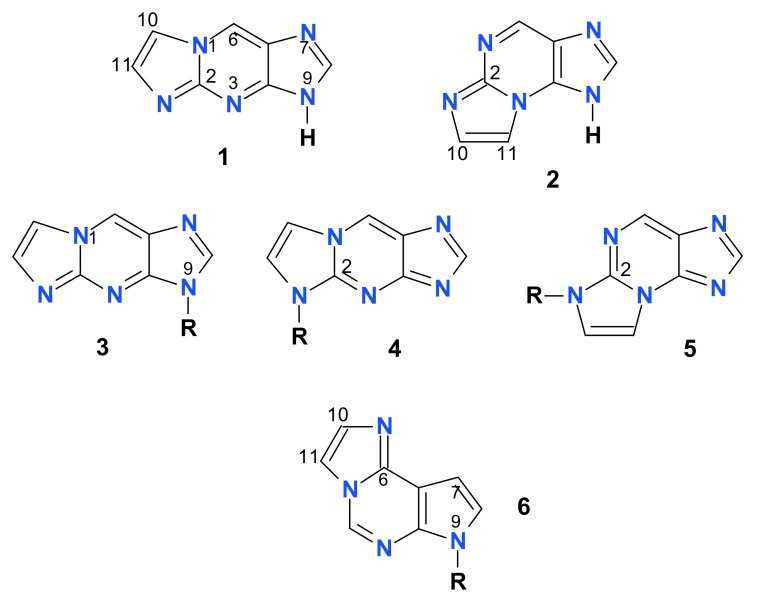
Structures of the nucleobase and nucleoside analogs investigated in this work: 1,N^2^-etheno-2-aminopurine (**1**), N^2^,3-etheno-2-aminopurine (**2**), and the ribosides of the ε2AP isomers (**3-5**, R = β-d-ribofuranosyl). Compound **6** is 1,N^6^-etheno-tubercidine (R = β-d-ribo-furanosyl). Only one tautomeric form of the bases is shown for simplicity. Note that the purine numbering is applied (except for the etheno-group atoms, numbered as 10 and 11).

The purpose of the present work is to examine some tri-cyclic amino-nucleobase analogs, in particular etheno derivatives of 2-aminopurine (ε2AP) and its riboside [23] and 7-deazadenosine (tubercidine [24,25], see Figure 1), as potential substrates and/or inhibitors of PNP, and possibly obtain in this way highly fluorescent compounds, useful for the future research, including study of enzyme-substrate and enzyme-inhibitor complexes. We have also extended spectral examination of the above-mentioned etheno-derivatives to include the respective tautomeric and ionic forms, the latter being important intermediates in enzymatic catalysis, with applications in the process of pharmaceutical design [26,27]. 

## 2. Results

### 2.1. Reaction of Chloroacetaldehyde with 2-Aminopurine and Its Riboside

2-Aminopurine riboside reacts rapidly with chloroacetaldehyde (CAA) at room temperature and weakly acidic pH, to give essentially one main product [23], identified as a linear adduct of CAA (1,N^2^-etheno-2-aminopurine-N^9^-β-d-riboside, (**3**)), readily crystallized from the neutralized reaction mixture. This product is moderately fluorescent in the visible part of the spectrum (see Table 1). The assignment of the ^1^H- and ^13^C-NMR signals is shown in Table 2. There are also traces of a second, highly fluorescent product, with spectral characteristics similar to the minor product of the reaction of 2-aminopurine with CAA (see next paragraph), but we were unable to isolate this compound in sufficient quantities. 

The reaction of 2-aminopurine (free base) with CAA was fairly rapid at pH ~ 4.5 (ca. 24 h), and gave two products (for the assignment of the NMR signals see Table 2). The major product, purified by re-crystallization, has been identified as 1,N^2^-etheno-2-aminopurine (1,N^2^-ε2AP (**1**)), with spectral (UV and fluorescence) characteristics very similar to those of the respective N^9^-riboside, described by Virta et al. [23]. Additionally, we have found a minor (~20%), but highly fluorescent product, identified as N^2^,3-etheno-2-aminopurine (N^2^,3-ε2AP, (**2**)), exhibiting excellent substrate properties towards PNP (see below). Both identified compounds revealed two pK_a_ values, one related to protonation (5–6.5) and the other to acidic dissociation of the imidazole proton (>8, see Table 1), thus confirming the identification of the products.

### 2.2. Properties of Two Isomers of Etheno-2-Aminopurine

**Figure 2:**
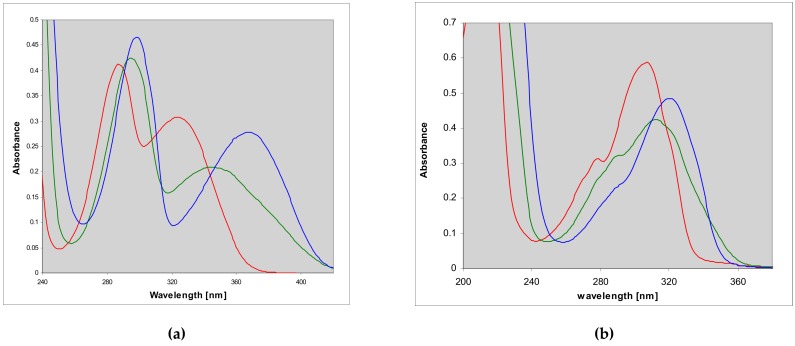
Electronic absorption spectra of (**a**) 1,N^2^-etheno-2-aminopurine (**1**) and (**b**) N^2^,3-etheno-2-aminopurine (**2**) in aqueous medium at various pH: red curves—pH 3; green—pH 7; blue—pH 11.

The electronic absorption spectra of the two 2-aminopurine etheno derivatives are presented below (Figure 2), and summarized in Table 1. The spectra of the linear isomer **1** under neutral conditions (phosphate buffer, pH 7) and in acid are strikingly similar to those of the N^9^-riboside **3**, published by Virta et al. [23], and are markedly red-shifted relative to analogous spectra of all adenine or guanine derivatives (cf. [21,22]). In basic media, the spectrum is shifted even more, showing maximum absorbance at 367 nm (Figure 2, Table 1). The minor, non-linear product (**2**) revealed low-energy absorption band near 300–320 nm. 

**Figure 3:**
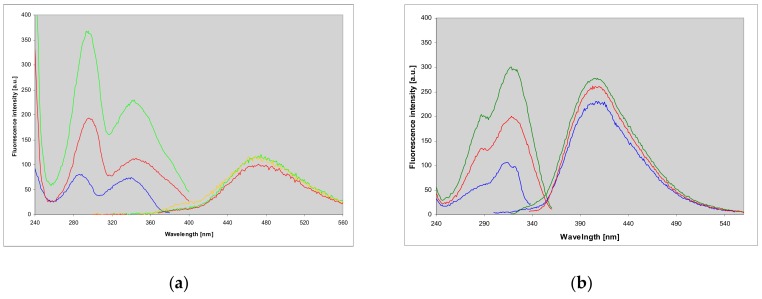
Fluorescence emission and corrected fluorescence excitation spectra of: (**a**) 1,N^2^-etheno-2-aminopurine (**1**), and (**b**) N^2^,3-etheno-2-aminopurine (**2**) in neutral aqueous solution. Emission spectra were measured with excitations at 310, 330 and 360 nm (green, yellow and red; left panel), and 310, 330 and 350 nm (green, blue and red, right panel), and excitation spectra with observations at 400, 440 and 480 nm (blue, green and red, left panel) and at 380, 400 and 440 nm (blue, green and red, right panel).

Both abovementioned products revealed moderate to intense fluorescence in neutral aqueous medium, centered at ca. 470 (1,N^2^-etheno-2-aminopurine (**1**)) and 405 nm (N^2^,3-etheno-2-aminopurine (**2**)), with yields approximately 0.18 and 0.73, respectively (see Figure 3 and Table 1). The fluorescence spectra of both isomers were to some extent excitation-dependent (Figure 3), and their fluorescence decays revealed non-exponential behavior (Table 1), the facts possibly related to protomeric equilibria in the ground state (see Discussion). 

**Figure 4:**
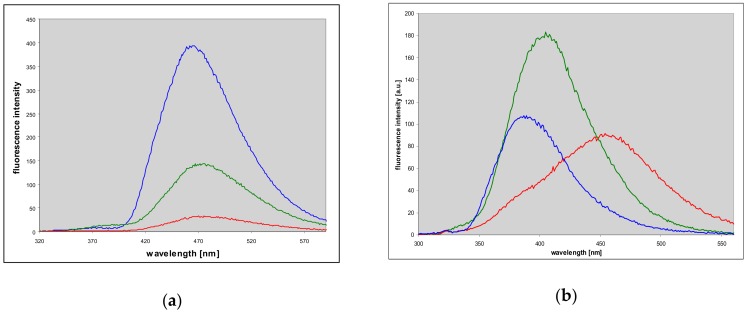
Fluorescence spectra of (**a**) 1,N^2^-etheno-2-aminopurine (**1**) and (**b**) N^2^,3-etheno-2-aminopurine (**2**) in aqueous medium at various pH: red curves, pH 3; green, pH 6.5 (**a**) and 7 (**b**); blue—pH 11.5. Excitation was at 300 nm (**a**) and 290 nm (**b**). The spectra of the ionic forms are virtually excitation-independent.

The ionic forms of the two tri-cyclic bases are also fluorescent (see Figure 4, below). Anionic and cationic forms of 1,N^2^-etheno-2-aminopurine (**1**) emit in the same region as the neutral molecule (460–470 nm), but with different yields (Figure 4a, Table 1). The largest Stokes’ shift is observed for the cationic species (9300 cm^−1^). The respective excitation spectra are virtually in line with UV absorption of each form. The anionic form of the non-linear isomer **2** is strongly fluorescent at 390 nm (Figure 4b, blue line), while the cationic form of this compound exhibits two-band fluorescence, with Stokes’ shift for the low-energy band exceeding 10,000 cm^−1^ (see Figure 4b, red curve).

### 2.3. Enzymatic Ribosylation of the Etheno-2-Aminopurine Isomers Using Various Forms of PNP

**Figure 5:**
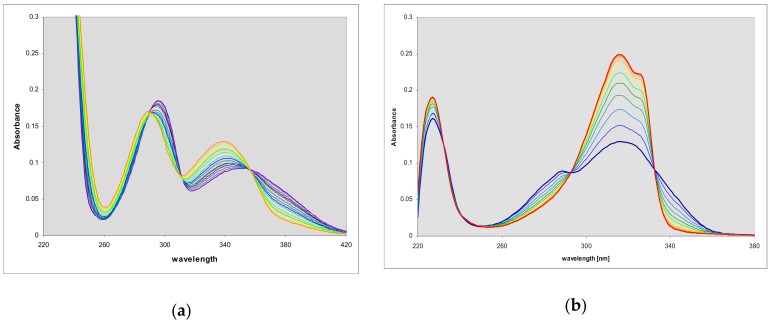
UV absorption changes observed during the enzymatic ribosylation of (**a**) 1,N^2^-etheno-2-aminopurine (**1**) with 0.5 mM R1P, catalyzed by PNP from *E. coli*; (**b**) enzymatic ribosylation of N^2^,3-etheno-2-aminopurine (**2**) by the same enzyme under identical conditions. Initial curves are in blue, and the final curves in red. Time intervals were 5 min for (**a**) and 2 min for (**b**).

**Figure 6:**
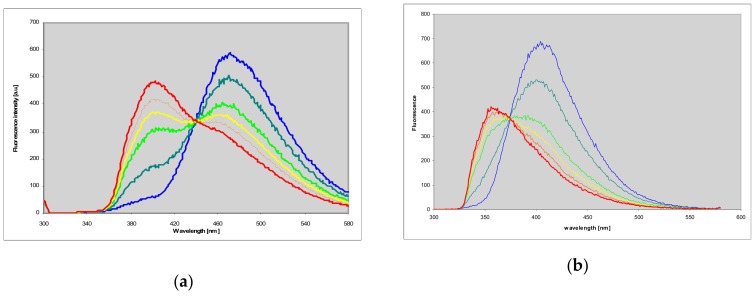
Fluorescence changes observed during the enzymatic ribosylation of (**a**) 1,N^2^-etheno-2-aminopurine (**1**) with 0.5 mM R1P, catalyzed by PNP from *E. coli*; (**b**) N^2^,3-etheno-2-aminopurine (**2**) by the same enzyme under identical conditions. Initial curves are in blue, and the final curves in red.

Both isomers of the etheno-2-aminopurine are substrates for PNP from *E. coli* in the reverse (synthetic) pathway with R1P as a ribosyl donor. Ribosylation of 1,N^2^-etheno-2-aminopurine (**1**) as well as N^2^,3-etheno-2-aminopurine (**2**) led to substantial changes in the UV absorption (Figure 5) and fluorescence (Figure 6) spectra, suggesting that the ribosylation sites may be different from the proton location in the respective base (the latter is possibly N^9^). We have also noted a striking similarity between UV absorption spectrum of the ribosylation products and the fluorescence excitation spectra of the minor tautomers of the respective bases, measured with observations at the blue edge of the emission spectrum (cf. Figure 2 and Figure 3). The ribosylation rate for the non-linear ε2AP isomer, N^2^,3-etheno-2-aminopurine (**2**), is ca. 40-fold higher than that for the linear isomer **1**, and comparable to the ribosylation rate of guanine, measured in the same conditions (Table 3).

Ribosides of 1,N^2^-etheno-2-aminopurin (**1**) as well as N^2^,3-etheno-2-aminopurine (**2**), generated using the *E. coli* PNP, were subjected to HPLC purification on a milligram scale, and their identification and properties are described in the next paragraph. 

Kinetic parameters of the synthetic (ribosylation) reaction, catalyzed by the wild-type and mutated forms of PNP, were determined using standard procedures, and are summarized in Table 3. There are some minor differences between wild-type enzymes (*E. coli* and calf PNP) and forms mutated in the active site, but without qualitative differences, observed previously for some purine analogs [21,22]. Generally, kinetic parameters for ribosylation of **2** by *E. coli* PNP and its mutated forms do not differ markedly from those determined earlier for natural purines [12], and the K_m_ values are close to 10 μM, hence comparable to those observed for guanine ribosylation under the same conditions. 

It may also be of interest that the trimeric calf spleen PNP, much more demanding in respect to substrate structures than the hexameric *E. coli* enzyme [12], ribosylates N^2^,3-etheno-2-aminopurine (**2**) with moderate rate (Table 3), but is apparently inactive towards the second (linear) isomer **1**. It may be of interest that N^2^,3-etheno-2-aminopurine (**2**) is fairly rapidly ribosylated by the calf PNP, mutated in the active site (N243D). But the ribosylation goes in an essentially similar way as with the *E. coli* PNP, that is, giving the identical single product (Table 3). 

### 2.4. Properties and Identification of the Enzymatically Produced Ribosides 

**Figure 7:**
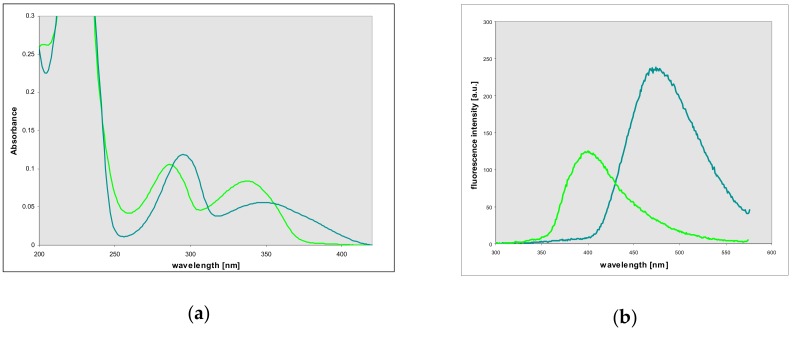
Comparison of (**a**) absorption and (**b**) fluorescence spectra of two ribosides of 1,N^2^-etheno-2-aminopurine (**3** and **4**), obtained chemically (**3**, dark green curves) and enzymatically **4**, using *E. coli* PNP as a catalyst (bright green curves). Spectra measured in phosphate buffer, pH 7. Fluorescence excitation is at 290 nm.

Reaction of 2-aminopurine riboside with chloroacetaldehyde gives N^9^-riboside (**3**) of the linear isomer of etheno-2-aminopurine (for the assignment of the ^1^H and ^13^C-NMR signals see Table 2), revealing spectral properties very similar to those of the respective base (see Section 3.2), but with a single emission band (465 nm) and a single decay time (Table 1). By contrast, the main product of enzymatic ribosylation of 1,N^2^-etheno-2-aminopurine, **4,** is characterized by the emission at 400 nm, single decay time, and the UV absorption shifted to the blue by over 20 nm (see Figure 5 and Figure 6, left panels, and Figure 7). This riboside undergoes protonation with pK_a_ ~6.3 (see Supplementary Materials, Figure S1). The compound has been subjected to purification using semi-preparative HPLC, and identified as 1,N^2^-etheno-2-aminopurine-N^2^-riboside ((**4**), see below).

**Figure 8:**
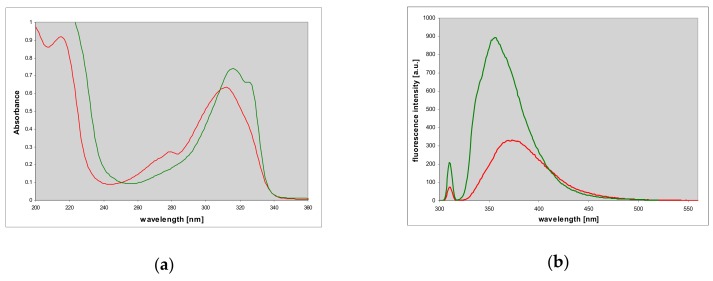
Electronic absorption (**a**) and fluorescence (**b**) spectra of the N^2^,3-etheno-2-aminopurine-N^2^-β-d-riboside (**5**) in aqueous media. Green lines: pH 7, red lines: pH 3. Fluorescence excitation was 310 nm.

The riboside produced enzymatically from the non-linear isomer of etheno-2-aminopurine, **5**, also differs spectrally from the parent base **2**. Its emission spectrum is shifted by ~45 nm to 355 nm, and UV absorption reveals fine structure (Figure 8). Fluorescence decay is mono-exponential, and decay time is lowered to ~2 ns, with yield ~0.29 (Table 1). The protonated form of the riboside is also strongly fluorescent, but we did not detect any traces of dual emission (as observed in the emission spectrum of the protonated base, see Figure 4), and the Stokes’ shift was moderate (Figure 8). We conclude that the photo-transformation, observed in the protonated base as two-band emission, is absent in the riboside.

The fluorescence decay times of all the ribosides **3**–**5**, both N^9^-ribo and N^2^-ribo, measured at pH > 7.5, were mono-exponential, in agreement with the view that no protomeric equilibrium is possible in the ground states of the ribosides, at least at their heteroaromatic moiety. Accordingly, fluorescence excitation spectra were in line with the UV absorption. Interestingly, the emission and excitation spectra of N^2^-ribosides **4**–**5** resemble those of the minor tautomers of both isomeric bases (see Section 3.2). This leads to tentative identification of the minor tautomers of both N^2^,3-ε2AP (**2**) and 1,N^2^-ε2AP (**1**) as N^2^H (see Discussion).

**Figure 9:**
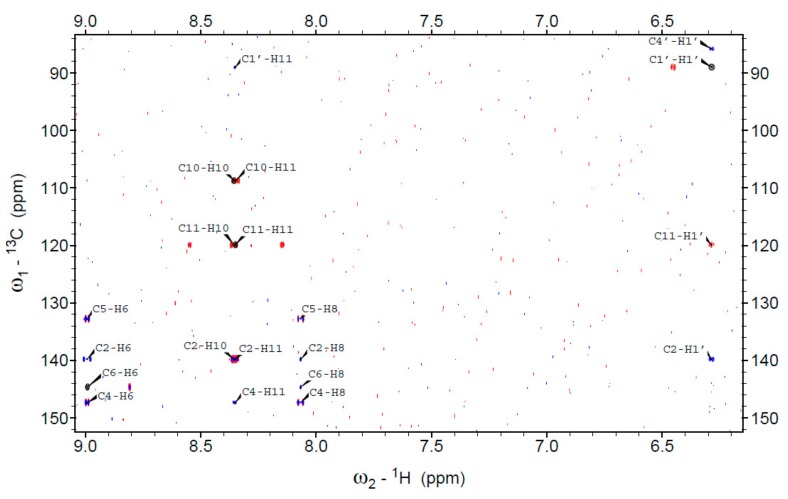
Aromatic and H(1′)/C(1′) ribose region of the contour plots of ^1^H, ^13^C correlation spectra of N^2^,3-etheno-2-aminopurine riboside (**5**) in DMSO-d_6_ (at ^1^H frequency of 500 MHz, at 25 °C) showing the cross-peaks between the ribose and nucleobase nuclei. Contours are plotted: for HSQC (black), for 8 Hz optimized HSQMBC (red), and for 2 Hz optimized HSQMBC (blue). All peaks are marked at the center of the multiplet and labeled according to the assigned nuclei (Table 2).

The ribosylation site of N^2^,3-etheno-2-aminopurine (**2**) via the enzymatic process was identified as N^2^ (for the assignment of the NMR signals in **5** see Table 2), based on the observation of non-vanishing three-bond scalar couplings between H1′ and both C2 and C11, as well as between H11 and C1′, in the ^1^H-^13^C HSQMBC spectrum (Figure 9). In the absence of other observable scalar couplings between the ribose and the base nuclei the position N^2^ is the only ribosylation site congruent with this coupling pattern. The ribosylation product of the linear isomer **1** was identified as 1,N^2^-etheno-2-aminopurine-N^2^-riboside (**5**), based on the observation of a coupling pattern fully analogous to that of the non-linear isomer.

The ribosides are fairly stable in solution, but we were unable to crystallize them due to small amounts obtained. They were stored as frozen in neutral aqueous solutions.

### 2.5. Phosphorolysis of The Ribosides with Various Forms of PNP

The highly fluorescent N^2^,3-etheno-2-aminopurine-N^2^-β-d-riboside (**5**), generated enzymatically (Section 3.3) is readily phosphorolyzed in the phosphate buffer by both *E. coli* and calf PNP. The reaction rates are in the case of *E. coli* enzyme comparable or even higher than that of guanosine phosphorolysis (Table 4), while those obtained for calf PNP are moderate. The observed spectral changes are reverse in respect to those presented of Figure 4 and Figure 5 for the synthetic process (see Supplementary Materials, Figure S2), and lead to very pronounced fluorogenic effect (not shown). Kinetic analysis revealed relative low K_m_ values for these reactions (Table 4), although they are somewhat higher than those for the synthetic reactions (Table 3).

We expect that the human PNP, which is quite similar to the calf enzyme [12], will also react with N^2^,3-etheno-2-aminopurine-N^2^-β-d-riboside (**5**), giving the highly fluorescent base **2** as a reaction product, with possible applications to analytical or clinical biochemistry. This point will be addressed in a separate paper. The ribosylated linear isomer, identified as 1,N^2^-etheno-2-aminopurine-N^2^-riboside (**4**) reacts much slower, and only with the *E. coli* PNP as a catalyst (Table 4).

### 2.6. Properties of 1,N^2^-Etheno-Tubercidine

Tubercidine (7-deazaadenosine) is a known antibiotic and an inhibitor of the bacterial (hexameric) forms of PNP [12]. It is also a substrate of some thermostable bacterial PNPs at higher temperatures [29,30]. The reaction of tubercidine and 2′-deoxytubercidine with chloroacetaldehyde gives the respective 1,N^6^-etheno derivatives in good yields [24,25]. 1,N^6^-Etheno-deoxytubercidine exhibits intense fluorescence at ~400 nm, similar to that of the analogous 1,N^6^-ethenoadenosine [25].

We have found that 1,N^6^-etheno-tubercidine (**6**) competitively inhibits phosphorolysis of purine nucleosides, catalyzed by the *E. coli* enzyme. The inhibition constant, K_i_ ~4.5 μM, is comparable to that of tubercidine itself [12], some formycin derivatives [31,32], and other good inhibitors of this enzyme [12]. Etheno-tubercidine (**6**) is therefore a good candidate to observe enzyme-ligand complexes by spectral methods, as shown below. No inhibition of the calf PNP was observed, at least in moderate concentrations (up to 50 μM) that we used. 

### 2.7. Fluorescence of Enzyme-Ligand Complexes

**Figure 10:**
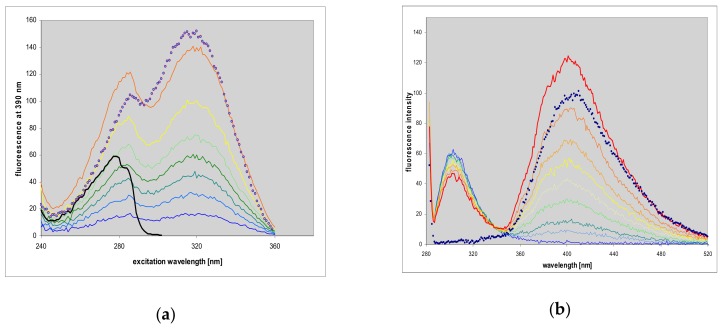

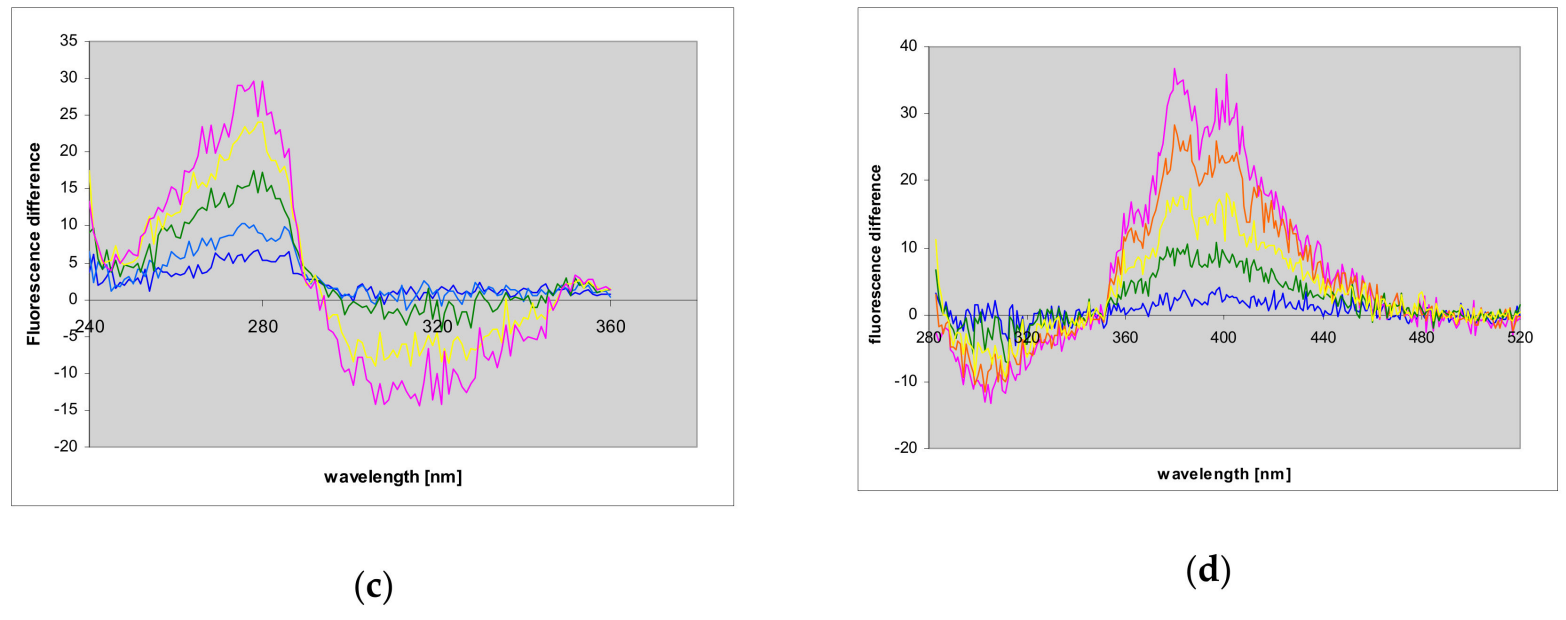
Changes in fluorescence excitation (**a**) and emission spectra (**b**), observed during the titration of *E. coli* PNP with N^2^,3-etheno-2-aminopurine (**2**). The excitation spectra were observed at 390 nm, and for emission spectra excitation was at 280 nm. Spectra of the free ligand (3.5 μM) are given for comparison (points). Excitation spectrum of the protein, monitored at 310 nm, is given in black. Conditions: 40 mM phosphate, pH 7, at 20 °C. Concentration of protein was 10.1 μM (per subunit); that of the titrant was 0 to 3.5 μM. Lower panels: Fluorescence difference spectra, calculated from data presented on panels (**a**) and (**b**), (**c**) difference excitation (observed at 380 nm), (**d**) difference emission spectra, excited at 275 nm. Substrate concentrations are 0.17, 0.69, 1.37, 2.36 and 3.31 μM.

Titration of the *E. coli* PNP with N^2^,3-etheno-2-aminopurine (**2**) in the presence of phosphate leads to moderate quenching of the protein fluorescence at 305 nm and formation of fluorescent complexes, visible both in emission and in excitation spectra (see Figure 10). Additionally, the fluorescence excitation spectra reveal fluorescence energy transfer (FRET) from the protein to the complexed ligand, since they show marked enhancement in the region 270–280 nm, where the tyrosine residues of the PNP molecule absorb (Figure 10). The fluorescence quantum yield of the bound ligand is very high and comparable to that of the free molecule (0.73, see Table 1), as evidenced by difference spectra (no negative contribution at the long-wavelength tail of the spectrum).

The respective difference spectra (Figure 10c,d) confirm moderate quenching of protein fluorescence (right-hand side), evidence of FRET (left), and appearance of the complex emission at 380–400 nm, revealing some fine structure, which is absent in the free ligand spectrum. We did not observe analogous complexes in the absence of phosphate, in spite of the low Michaelis’ constants obtained for the synthetic reaction (Table 3).

**Figure 11:**
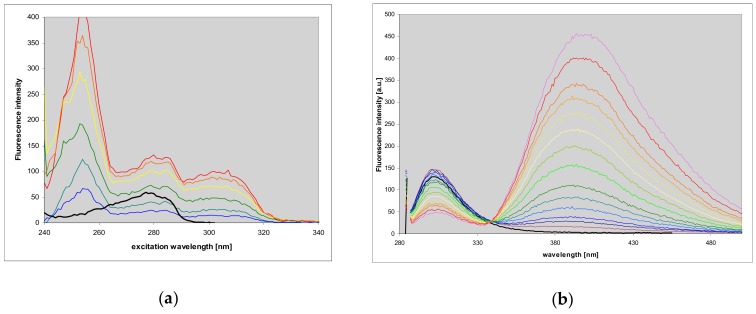

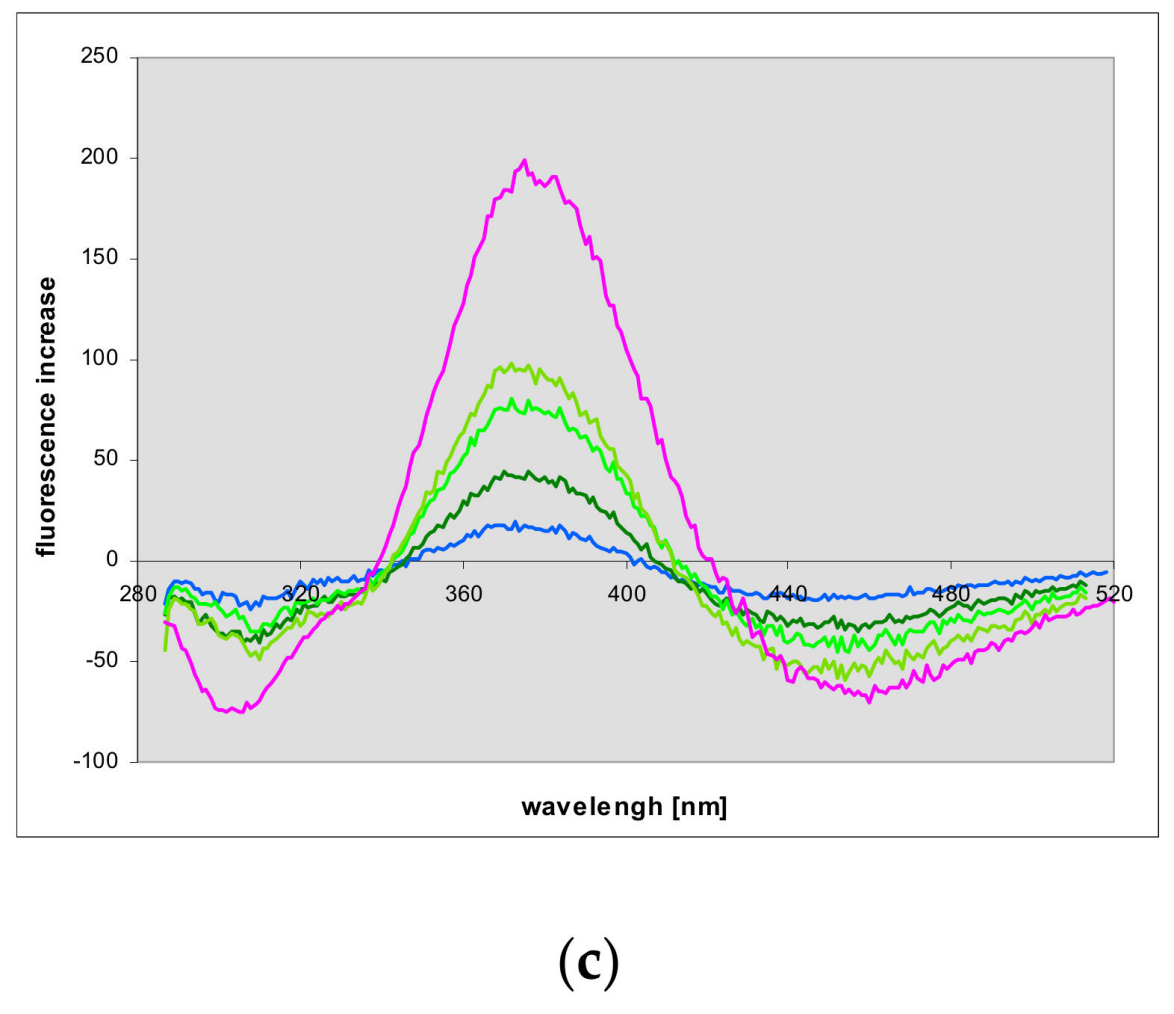
Changes in fluorescence excitation (**a**) and emission spectra (**b**), observed during the titration of *E. coli* PNP with 1,N^6^-etheno-tubercidine (**6**). Emission spectra were recorded with excitation at 280 nm, excitation spectra were observed at 380 nm. Conditions: 40 mM phosphate buffer, pH 7, at 20 °C. The concentration of protein was 11 μM per subunit; that of 1,N^6^-etheno-tubercidine was (0–18.5 μM). Black curves refer to pure protein emission or excitation (observed at 310 nm); (**c**) fluorescence difference spectra of 11 μM *E. coli* PNP, titrated with 1,N^6^-etheno-tubercidine (1.9, 3.7, 5.4, 7.1 and 18.5 μM), calculated from data in panel (**b**).

Etheno-tubercidine (**6**) forms somewhat similar fluorescent complexes with the *E. coli* PNP. As shown below, FRET from the protein to the ligand is evident in the excitation spectra (Figure 11), and the ligand fluorescence blue shifted from 415 to ca. 390 nm (Figure 11b,c). In this case more than 60% of protein fluorescence is quenched when protein binding sites are saturated (cf. Figure 11). Very small changes in the excitation spectra suggest that the ligand is bound to the protein as a neutral species; but some irregularities in the difference spectra (Figure 11c) may indicate that the binding sites in the hexameric PNP molecule are not equivalent, but this conclusion needs verification.

## 3. Discussion

### 3.1. Fluorescent Isomers of the Etheno-2-Aminopurine

Vinyl chloride, a known chemical mutagen and carcinogen, acts as a modifier of nucleobases, in particular, of adenine and guanine moieties [14,15], which upon this chemical modification change the respective coding properties, leading to the mutagenic effect [33,34]. In addition some of the bases modified in this way exhibit additionally marked fluorescence [1,2,3,4], which make them good candidates for fluorescent probes in enzymological research. 

**Figure 12:**
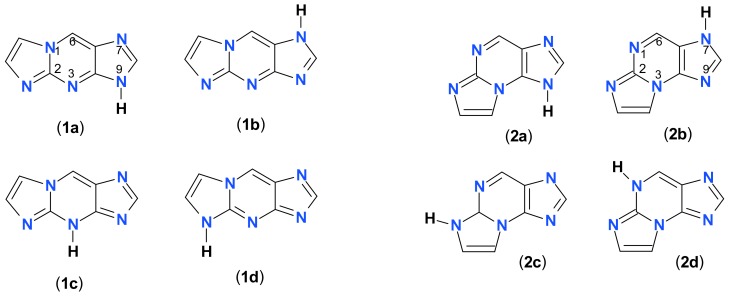
Possible tautomeric structures of two isomers of etheno-2-aminopurine: 1,N^2^-etheno-2-aminopurine (**1a**–**d**), N^2^,3-etheno-2-aminopurine (**2a**–**d**).

We found two isomeric products of the reaction of CAA with 2-aminopurine, both revealing intense fluorescence in aqueous medium. In both cases, fluorescence was excitation-dependent and decays were non-exponential. The most likely interpretation of this fact is the N^9^H-N^7^H tautomerism, confirmed for some purines and analogs [35,36,37], and suggested for 1,N^6^-ethenoadenine [21], but other tautomeric forms, like N^2^H or N^3^H, cannot be excluded (see Figure 12). The similarity between properties of the minor tautomers and N^2^-ribosides strongly suggests participation of the N^2^H protomers **1d**, **2c**. 

Large Stokes’ shifts observed for the cationic species of both isomers suggests photo-transformations. Such photo-transformations (e.g., excited-state proton transfer and a resulting photo-tautomerism) are not infrequent among the fluorescent purine analogs and derivatives [38].

### 3.2. Enzymatic Syntheses of the Tri-Cyclic Ribosides and Their Properties

In this and preceding papers [21,22], we have described substrate and inhibitor properties of several tri-cyclic nucleobase analogs towards the enzyme purine-nucleoside phosphorylase. Many of the investigated compounds, in particular, etheno-adenosine and one isomer of etheno-guanosine were found to be excellent substrates for the bacterial (*E. coli*) type of PNP, and the respective bases were easily ribosylated in the reverse process [21,22]. Enzymatic ribosylation of the tri-cyclic nucleobase analogs and similar compounds leads to non-typical ribosides, which are nevertheless good substrates for PNP, as shown previously for the N^6^-ribosylated 1,N^6^-ethenoadenine [21], and analogous isoguanine derivatives [22]. 

Both isomers of etheno-2-aminopurine (**1** and **2**) are ribosylated using R1P as a ribose donor and the *E. coli* PNP as a catalyst, but the ribosylation site is N^2^, rather than N^9^. Somewhat similar ribosylation pattern was previously reported for etheno-adenine [21] and etheno-isoguanine [22], but in both cases different enzyme forms led to different ribosylation products. By contrast, the linear isomer of etheno-guanine was rapidly ribosylated on the “canonical” N^9^ nitrogen [22]. 

This ambiguity in the ribosylation sites in enzymatic reactions, catalyzed by PNP, was also previously observed in 8-azapurines [39,40], and probably results from the plasticity of the active site of this class of enzymes. For example, in the X-ray crystal structure of hexameric PNP from *H. pylori*, bound to an inhibitor formycin A (8-aza-N^9^-deazaadenosine), both the standard (*anti*) and non-standard (*syn*) conformations of the inhibitor were revealed [41]. In another study, acyclo-guanosine, an inhibitor of mammalian PNPs, with the acyclic chain bound to N^7^ position of the purine base, was found in the inverted (‘up-side-down’) position in the active site of calf PNP, with the chain located in the place normally occupied by the ribose [42]. Finally, N^3^-β-d-ribofuranosyladenine and N^3^-β-d-ribofuranosyl-hypoxanthine, with sugar moiety attached to the N_3_ position of the base, were found to be non-conventional substrates of purine nucleoside phosphorylase from *E. coli* and calf [43].

Some of the fluorescent tri-cyclic ribosides are also moderate to good substrates of mammalian PNPs, known to be homologous to human enzymes [12]. This makes them potential indicators of PNP activity in biological or clinical samples. As an illustration, we have recently shown that human erythrocytic PNP activity can be measured fluorimetrically in 1000-fold diluted hemolysates using the N^6^-ribosylated 1,N^6^-ethenoadenine as an artificial substrate [44]. We expect that the new compounds presented in this work, particularly the ribosides of N^2^,3-etheno-2-aminopurine (**2**), will exceed in sensitivity other fluorescent indicators of PNP activity (to be published elsewhere).

### 3.3. Fluorescent Complexes

Various forms of PNP form fluorescent complexes with purines and their analogs [17]. Long ago Porter et al. [45] reported a fluorescent complex of calf PNP with guanine, ascribing its fluorescence to the anionic form of the ligand. Later investigations with fluorescent 8-azaguanine derivatives and calf PNP [46,47] instead suggested neutral ligand as the emission source. The *E. coli* PNP is an interesting object of this kind of experiments since its molecule does not contain tryptophan and its native fluorescence is located near 305 nm [12], allowing easy observations of emission of the complexes, as illustrated by Kierdaszuk et al. [31,32]. In the latter case, it was possible to identify individual protomeric forms of the ligand formycin A (8-aza-N^9^-deazaadenosine), when bound to the PNP molecule. 

Somewhat similar fluorescent complexes and FRET were previously observed with *E. coli* PNP complexed with formycin A and its *N*-methyl derivatives as ligands [31]. Now, we present evidence for the highly fluorescent base-enzyme complexes, observed in the presence of phosphate. We did not observe analogous complexes in the absence of phosphate, in spite of the low Michaelis’ constants obtained for the synthetic reaction (Table 3). This is in line with the fact that for both mammalian and bacterial PNPs, due to complex mechanisms of catalysis exhibited by these two enzyme families, K_m_ does not describe the affinity of most substrates, e.g., [12,16,48] adequately. This also suggests that, in agreement with previous mechanistic considerations and stabilization pattern exhibited by substrates [49], the purine base is the only *E. coli* PNP substrate that cannot bind to the enzyme molecule in the absence of either a second substrate (in this case R1P) in the synthetic reaction, or phosphate, a substrate in the reverse phosphorolytic path, in the latter case forming a so-called dead-end complex.

Previous papers have shown that the *E. coli* PNP molecule can selectively bind individual tautomeric forms of some ligands [31,32]. At present, we cannot identify which of possible tautomeric structures is bound, but the minor tautomer, visible in solution spectra as an inflection, and postulated to be the N^2^H protomer **2c**, is evidently not responsible for the above changes since the emission spectra of the complex differ markedly from those of the N^2^-riboside **5** (cf. Figure 10 and Figure 5). Our interpretation of the presented data is that N^7^ or N^9^ are likely locations of the proton in the complex.

## 4. Materials and Methods 

### 4.1. Chemicals

2-Aminopurine (2AP), chloroacetaldehyde (CAA) and 7-methylguanosine (m^7^Guo) were from Sigma-Aldrich (St. Louis, MO, USA). Tubercidine (7-deazaadenosine) was a gift from Dr. Janusz Stępiński from the University of Warsaw (Warsaw, Poland). 2-Aminopurine-N^9^-β-d-riboside was from Santa Cruz Biotechnology (Dallas, Texas, USA).

The ribose source for enzymatic ribosylation, α-d-ribose-1-phosphate (R1P) has been prepared enzymatically as a 100 mM solution from 7-methylguanosine phosphorolysed by calf PNP, as previously described [33,50] and kept frozen.

Etheno-derivatives of 2-aminopurine (ε2AP) were obtained as follows: 2-aminopurine (2AP, 1 g, 7.4 mmoles) was dissolved in 30 mL of ~0.1 M acetic acid. After adjusting pH to ~4 by sodium bicarbonate, the mixture was treated with 0.5 mL CAA (50% aqueous solution), for ca. 24 h at room temperature, and warming at the end to ~40 °C for ca. 30 min. HPLC analysis showed the disappearance of >90% 2AP. The solution was neutralized by sodium bicarbonate and left in a refrigerator for 24 hours. The major product, identified as 1,N^2^-etheno-2-aminopurine (1,N^2^-ε2AP, (**1**))*,* crystallized in the form of plates (~400 mg, 2.5 mmoles, slowly darkening in the air; overall yield ~35%). The mother liquors were subjected to semi-preparative HPLC to give ca. 50 mg (5%) of an additional, minor but highly fluorescent product, identified as N^2^,3-etheno-2-aminopurine (N^2^,3-ε2AP (**2**)). This product was concentrated and kept frozen. MS: comp. **1**: *m/z* = 160.0617, comp. **2**: *m/z* = 160. 0619; calculated for C_7_H_5_N_5_ + H: 160.0612.

**Figure 13:**
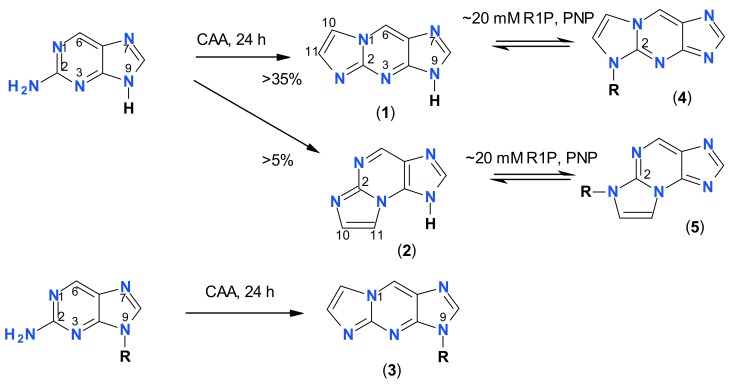
Synthesis of 2-aminopurine etheno-derivatives and their ribosides (R = β-d-ribofuranosyl; CAA = chloroacetaldehyde, R1P–α-d-ribose-1-phosphate; PNP = purine-nucleoside phosphorylase).

The etheno-derivative of 2-aminopurine-N^9^-β-d-riboside (Figure 13) was prepared according to Virta et al. [23], and crystallized from the neutralized reaction mixture. Its structure was confirmed by NMR data, which were in agreement with the published results [23], and by MS: comp. **3**: *m/z* = 292.10385, calculated for C_12_H_13_N_5_O_4_ + H: 292.10403.

The reaction of chloroacetaldehyde with tubercidine (7-deazaadenosine) was carried out as previously described [24], with the modification that an aqueous CAA was applied instead of the distilled reactant. The product purified by semi-preparative HPLC in milligram quantities (approximate yield > 60%). The spectral parameters were in agreement with those previously published [24]. See Supplementary Materials for more details.

Product separation and purification were performed by HPLC on a UFLC system from Shimadzu (Kyoto, Japan) equipped with UV (diode-array) detection at 260, 280 and 315 nm, and a fluorescence detector. The column used was a Kromasil reversed-phase, semi-preparative C-18 column (250 × 10 mm, 5-μm particle size). Elution was initially (10–15 min) isocratic, followed by a water-methanol gradient (usually 10–30% methanol for 40 min, see Supplementary Materials for details). All buffers were of analytical grade and showed no fluorescence background.

### 4.2. Spectral Measurements

Fluorescence spectra were measured on a Varian Eclipse instrument (Varian Corp., Palo Alto, CA, USA), and UV absorption kinetic experiments were performed on a Cary 5000 (Varian Corp., Palo Alto, CA, USA) thermostated spectrophotometer. Fluorescence yields were determined relative to tryptophan (0.15) or 1,N^6^-ethenoadenosine in water (0.56, see ref. [2]). Emission spectra were measured in semi-micro cuvettes, pathlength 4 mm, to diminish the inner-filter effect. Typical spectral resolution was 2.5 nm. The ionization constants (pK_a_ values) were determined spectrophotometrically using 20–50 mM phosphate and/or acetate buffers.

Fluorescence decays were measured and analyzed using a FluoTime 200 lifetime fluorometer (PicoQuant GmbH, Berlin, Germany), equipped with an R3809U-50 microchannel-plate photo-multiplier (MCP-PMT, Hamamatsu, Japan), with 280 nm excitation by sub-nanosecond pulsed LED, as previously described [50].

NMR measurements were run in DMSO-d_6_ at 25 °C on an Avance III HD 800 MHz spectrometer Bruker (Bruker BioSpin AG, Fällanden, Switzerland) equipped with a cryogenically-cooled triple resonance (HCN) probe (the sample identified as the N^2^-riboside of 1,N^2^-etheno-2-aminopurine (**3**)) and on a Bruker Avance III HD 500 MHz spectrometer equipped with a room-temperature triple resonance (HCN) probe. For all samples, the following spectra were acquired: a standard 1D proton spectrum, a ^1^H, ^1^H CLIP-COSY [51] and a gradient-selected ^1^H, ^13^C HSQC [52]. Phase-sensitive gradient-selected HMBC spectra tuned for 2 and 8 Hz J couplings were acquired for the N^2^ riboside of 1,N^2^-etheno-2-aminopurine (**3**), while gradient-selected ^1^H, ^13^C HSQMBC ([53], modified by using hard pulses) were run for all other samples—tuned for optimal detection of 12 Hz J couplings in the case of 1, N^2^-etheno-2-aminopurine or two separate experiments tuned for 2 Hz and 8 Hz J couplings otherwise. ^1^H chemical shifts were referenced by the field-locked substitution method using a (less than) 1% sample of TMS in DMSO-d_6_, and ^13^C chemical shifts were referenced using the unified chemical shift scale [54]. The 2D spectra were processed using the TopSpin 3.6.1 software package (Bruker) and inspected by the Sparky program [55] with manual peak-picking.

### 4.3. Mass Spectrometry

The structure and purity of the new compounds **1**–**2** and **4**–**5** were confirmed using the high-resolution mass spectrometry with positive electrospray ionization HRMS (+) ESI. Mass spectra were recorded on a Thermo Scientific QExactive spectrometer (Thermo Fisher Scientific, Waltham, MA, USA). Spray voltage was 3800 V, capillary temperature 320 °C.

### 4.4. Enzymes and Enzymatic Reactions

Recombinant *E. coli* PNP, calf spleen PNP, and their mutated forms were expressed in *E. coli* and purified according to the procedures described earlier [56,57]. Enzyme concentrations were calculated per monomer.

Enzymatic ribosylation reactions were carried out in 1 mL cuvettes (pathlength 4 mm) in ~50 mM HEPES buffer, pH 7.3, using 0.5 mM R1P as a ribose source. Reactions were followed fluorimetrically (see Section 2.4). On a larger scale (2–4 mg), the reactions were run in Eppendorf tubes, volume 2–3 mL, using either R1P (3–10 mM) or 7-methylguanosine as ribose sources. Products were purified by semi-preparative HPLC, concentrated and the final solutions kept frozen. Their structure has been confirmed by NMR (see Section 2.4 and Supplementary Materials) and by MS: comp. **3**: *m/z* = 292.10385, comp. **4**: *m/z* = 292.10400, comp. **5**: *m/z* = 292.10398, calculated for C_12_H_13_N_5_O_4_ + H: 292.10403. Phosphorolysis reactions were run in 40–50 mM phosphate buffer, pH 7.0 or 6.5, and followed spectrally or fluorimetrically. Kinetic parameters were calculated by standard methods.

## 5. Conclusions 

We have shown that 2-aminopurine easily reacts with aqueous chloroacetaldehyde to give two fluorescent, isomeric products **1** and **2**. Both products are substrates for the bacterial (*E. coli*) purine nucleoside phosphorylase, but in both cases the ribosylation site has been found to be N^2^ rather than N^9^. The new ribosides are fluorescent and potentially useful as fluorescent probes. The antibiotic tubercidine (7-deazaadenosine) also reacts with aqueous chloroacetaldehyde to give the fluorescent product **6**. This product is an inhibitor of the *E. coli* PNP and forms fluorescent complexes with the enzyme.

## Figures and Tables

**Table 1:** Ionization constants (pK_a_ values, determined spectrophotometrically) and spectral parameters for neutral and ionic forms of the investigated compounds (n—neutral form; c—cation; a—anion)**.** Nd = not determined.

Compound	pK_a_	form (pH)	UV Absorption λ_max_ (nm) ε_max_	Fluorescence λ_max_ (nm) φ	τ [ns]
1,N^2^-etheno-2-aminopurine (**1**)	5.6; 8.2	n(7)	348 2560	472 0.18	6.9; 10.3
		c(3)	285 5050	467 ~0.04	Nd
		a(11)	367 3500	463 ~0.40	Nd
N^2^-β-d-Ribofuranoside (**4**)	6.3	n(8)	338 4260	406 0.73	Nd
		c(3)	326 Nd	406 0.73	Nd
N^9^-β-d-Ribofuranoside (**3**)	-	n(8)	295 5700 ^1^	463 0.14	Nd
		c(3)	318 6120	465 0.06	Nd
N^2^,3-etheno-2-aminopurine (**2**)	5.0; ~9.5	n(7)	315 ~5600	406 0.73	3.8; 8.5
		c(2.5)	306 ~7700	455 ~0.30	Nd
		a(12)	320 ~6700	390 ~0.40	Nd
N^2^-β-d-Ribofuranoside (**5**)	6.15	n(8)	315 ~10,700	357 0.29	2.15
		c(3)	310 ~9400	373 ~0.15	Nd
1,N^6^-ethenotubercidine (**6**)	5.2	n(7)	288 5800 ^2^	415 0.53 ^2^	Nd
		c(3)	281 8700	415 Nd	Nd

^1^ Data from Virta et al. [23] and ^2^ from Seela et al. [25] for 2’-deoxytubercidine.

**Table 2:** ^1^H- and ^13^C-NMR chemical shifts in parts per million (±0.001) of the etheno derivatives of 2-aminopurine (ε2AP) and their β-d-ribosides (see Materials and Methods for details). Chemical shift labels follow the naming convention of [28], extended for the etheno protons (see Figure 1).

Nucleus	1,N^2^-ε2AP (1)	1,N^2^-ε2AP- N^9^-ribofuranoside (3)	1,N^2^-ε2AP- N^2^-ribofuranoside (4)	N^2^,3-ε2AP (2)	N^2^,3-ε2AP- N^2^-ribofuranoside (5)
H6	9.445	9.541	9.246	8.879	8.992
H8	8.513	8.768	8.403	8.408	8.067
H10 ^1^	7.624	7.661	7.987	8.115	8.356
H11 ^1^	7.895	7.931	8.092	7.719	8.351
H1′	--	5.969	6.212	--	6.286
H2′	--	4.617	4.418	--	4.415
OH2′	--	5.531	5.592	--	5.612
H3′	--	4.189	4.152	--	4.177
OH3′	--	5.217	5.343	--	5.249
H4′	--	3.962	3.985	--	4.010
H5′/H5′′	--	3.581/3.690	3.601/3.694	--	3.630/3.733
OH5′	--	5.090	5.162	--	5.210
C2	147.470	146.868	141.394	147.740	139.817
C4	152.793	150.122	165.789	143.330	147.377
C5	126.637	127.994	134.105	116.873	132.921
C8	149.972	149.029	167.161	142.398	155.724
C10 ^1^	133.727	134.305	111.629	107.780	108.777
C11 ^1^	110.641	111.080	118.837	133.172	119.946
C6	125.239	127.102	123.368	138.144	144.668
C1′	--	87.495	88.07	--	89.037
C2′	--	73.529	74.439	--	74.969
C3′	--	70.568	70.471	--	70.151
C4′	--	85.706	85.761	--	85.798
C5′	--	61.603	61.443	--	61.059

^1^ Due to the possibility of non-vanishing five-bond ^1^H,^13^C couplings in aromatic structures the assignment of the H10/C10 and H11/C11 pairs is tentative and may be reversed.

**Table 3:** Kinetic parameters for the enzymatic ribosylation of selected etheno-purine derivatives in 40 mM HEPES buffer, pH 7, by α-d-ribose-1-phosphate, using various forms of PNP (wt = wild type; nr—no reaction detected; nd—not determined). Standard errors are estimated to be ~15%.

Substrate	Enzyme (PNP Source)	K_m_ (μM)	V_max_ (Relative) ^1^	Ribosylation Site
1,N^2^-etheno-2-aminopurine (**1**)	calf-wt	nr	< 0.1	-
“	calf—N243D	nd	traces	nd
“	*E. coli*-wt	<10	~1	mostly N^2^
N^2^,3-etheno-2-aminopurine (**2**)	calf-wt	~8	~5	predominantly N^2^
“	calf—N243D	110	29	predominantly N^2^
“	*E. coli*-wt	11	20	predominantly N^2^
“	*E. coli*—D204N	12	30	predominantly N^2^
“	*E. coli*—S90A	~20	23	predominantly N^2^

^1^ Relative to guanine ribosylation rate under identical conditions (set as 100).

**Table 4:** Kinetic parameters for the enzymatic phosphorolysis of selected etheno-2-aminopurine **ribosides** in 40 mM phosphate buffer, pH 7, using various forms of PNP. Standard errors are estimated to be ~ 20%; nr = no reaction observed.

Substrate	Enzyme ^2^	K_m_ (μM)	V_max_ (relative) ^1^
1,N^2^-etheno-2-aminopurine-N^9^-β-d-riboside (**3**)	*E. coli* PNP-wt	nr	<1
-	*calf* PNP-wt	nr	<0.1
1,N^2^-etheno-2-aminopurine-N^2^-β-d-riboside (**4**)	*E. coli* PNP-wt	47	4
-	*calf* PNP-wt	nr	<0.1
N^2^,3-etheno-2-aminopurine-N^2^-β-d-riboside (**5**)	*E. coli* PNP-wt	~ 20	115
-	*calf* PNP-wt	4.6	3

^1^ Relative to V_max_ of guanosine phosphorolysis under the same conditions (=100); ^2^ wt = wild type.

## Supplementary Materials

The Supplementary Materials are available online.
