# Supplementary material for: Tricyclic Nucleobase Analogs and Their Ribosides as Substrates and Inhibitors of Purine-Nucleoside Phosphorylases III. Aminopurine Derivatives
Source: Molecules. 2020 Feb 5;25(3):681. doi: 10.3390/molecules25030681 (PMC7037862; doi:10.3390/molecules25030681)
Supplement: Supplementary file 1 [file molecules-25-00681-s001.pdf]

molecules

Supplementary Materials

Chemical procedures:

1,N[2]-etheno-2-aminopurine (**1**) and N[2],3-etheno-2-aminopurine (**2**): 2-aminopurine (2AP, 1 g, 7.4 mmoles) was dissolved in 30 mL of ~0.1 M acetic acid. After adjusting pH to ~4 by sodium bicarbonate, the mixture was treated with 0.5 mL CAA (50% aqueous solution), for ca. 24 hrs at room temperature, and warming at the end to ~40 °C for ca. 30 min. The solution was neutralized by sodium bicarbonate and left in a refrigerator for 24 hrs. The major product, identified as 1,N[2]-etheno-2-aminopurine (1,N[2]-ε2AP, (**1**)), crystallized in the form of plates (~400 mg, 2.5 mmoles, slowly darkening in the air; overall yield ~35%). The remaining liquors were subjected to semi-preparative HPLC. The column (see section 5.1) was eluted with water for 13 min, followed by 40 min of water-methanol gradient 5-25%. Flow rate was 3 ml/min and the retention time of **1** was 16 minutes and of **2** 32 minutes. The product **2** (~10 mg) was evaporated to dryness and **1** was discarded.

1,N[6]-etheno-tubercidine (**6**) 5 mg of tubercidine was dissolved in ~2 mL of 0.1 M acetic acid. 0.2 mL of 50% aqueous CAA has been added and after half an hour pH adjusted to ~4 by sodium bicarbonate. Reaction proceeded for 24 hours at ca. 30 °C, the product was purified using semi-preparative HPLC as described under Materials and Methods. Eluent was water, 13 min, followed by 40 min of water-methanol gradient 0-60%. Flow rate was 3 ml/min and the retention time 30-32 minutes. The product was concentrated and kept frozen.

Enzymatic procedures:

1,N[2]-etheno-2-aminopurine-N[2]-riboside (**4**): 7 mg of **1** has been dissolved in 1 mL of ca. 0.1 M acetic acid. After neutralization with sodium bicarbonate, 0.5 mL of 100 mM r1P solution was added, followed by the *E. coli* PNP to final concentration of ~4 µM. The reaction was run for 24 hrs at room temperature. After this perion, the resultant mixture was again acidified to pH 4.5 and the product separated by semi-preparative HPLC (see section 5.1). Eluent was water, 10 min, followed by 40 min of water-methanol gradient 6-15%. Flow rate was 3 ml/min and the retention time of **5** was 18-21 minutes, while the unreacted staring substance **1** was eluted within first 8-10 minutes. The product (ca. 3 mg) was evaporated to dryness and kept frozen.

N[2],3-etheno-2-aminopurine-N[2]-riboside (**5**): 2 mg of **2** has been dissolved in 1 mL of ca. 0.1 M acetic acid. The mixture was neutralized with sodium bicarbonate and treated with 0.3 mL of 100 mM R1P. PNP from *E. coli* has been added to final concentration ~2 µM and the reaction allowed to run for ca. 24 hrs at room temperature. The reaction progress was followed fluorimetrically (excitation 290 nm), until the fluorescence maximum shifted to 355 nm. After finishing, pH was adjusted to 4 with acetic acid, and the medium subjected to semi-preparative HPLC (see Materials and Methods). Eluent was water, 13 min, followed by 40 min of water-methanol gradient 11-16%. Flow rate was 3 ml/min and the retention time 40-45 minutes. The product was concentrated and kept frozen.

Titration data for the new ribosides **4** and **5** are given below. These compounds are synthesized on a milligram scale and kept frozen in concentrated aqueous solutions.

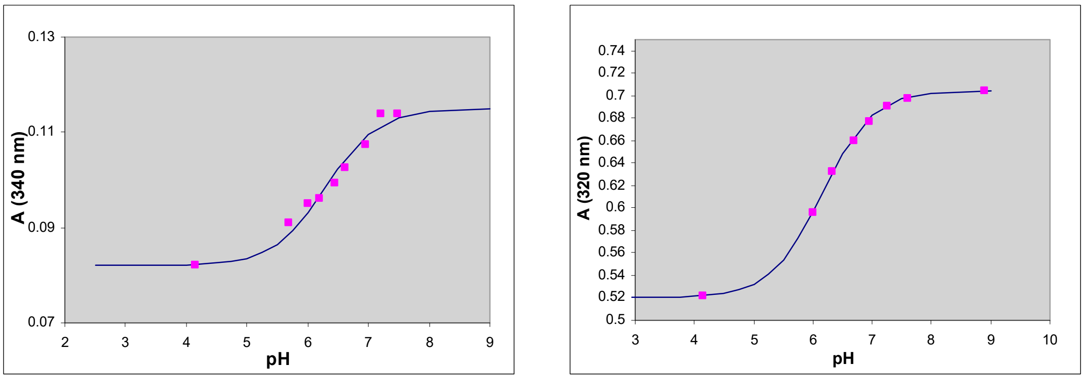

(**a**) (**b**)

Figure 1S. Spectrophotometric titration of 1,N²-etheno-2-aminopurine-N²-riboside (**4,** panel **a**) at 340 nm and N²,3-etheno-2-aminopurine-N²-riboside (**5,** panel **b**) at 320 nm. The fitted pKₐ values are 6.3 and 6.15, respectively.

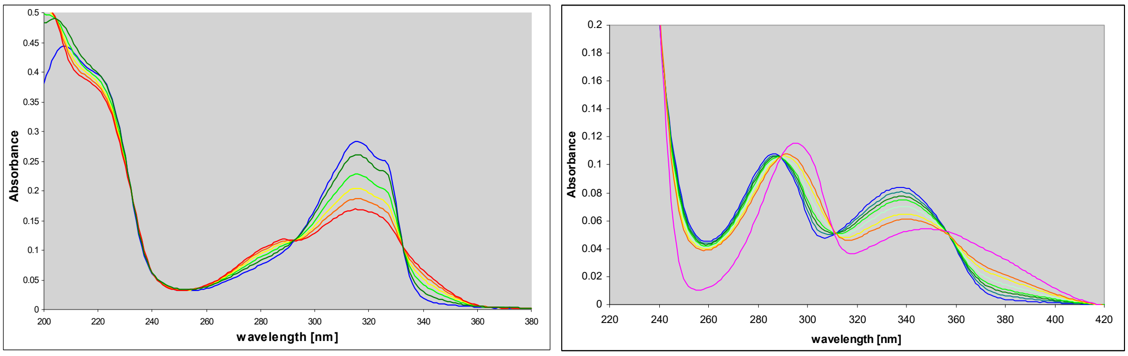

(**a**) (**b**)

Figure 2S. Spectrophotometric observation of the phosphorolysis of 30 µM N²,3-etheno-2-aminopurine-N²-riboside (**5,** panel a) and 1,N²-etheno-2-aminopurine-N²-riboside (**4,** panel **b**) by PNP from *E. coli*. The reaction was carried out in 40 mM phosphate buffer, pH 7, at 25° C.

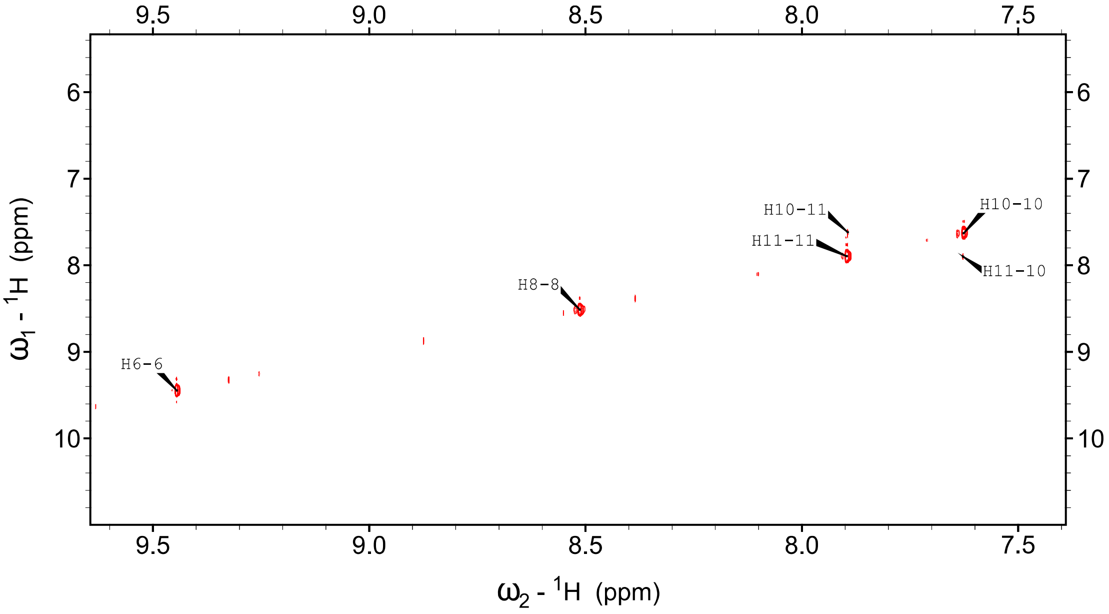

Figure 3S. Contour plot of the ^{1}H, ^{1}H COSY spectrum of 1,N^2-etheno-2-aminopurine (**1**) in DMSO-d$_6$ (at ^{1}H frequency of 500 MHz, at 25 °C). All peaks are marked at the center of the multiplet and labeled according to the assigned nuclei (Table 2).

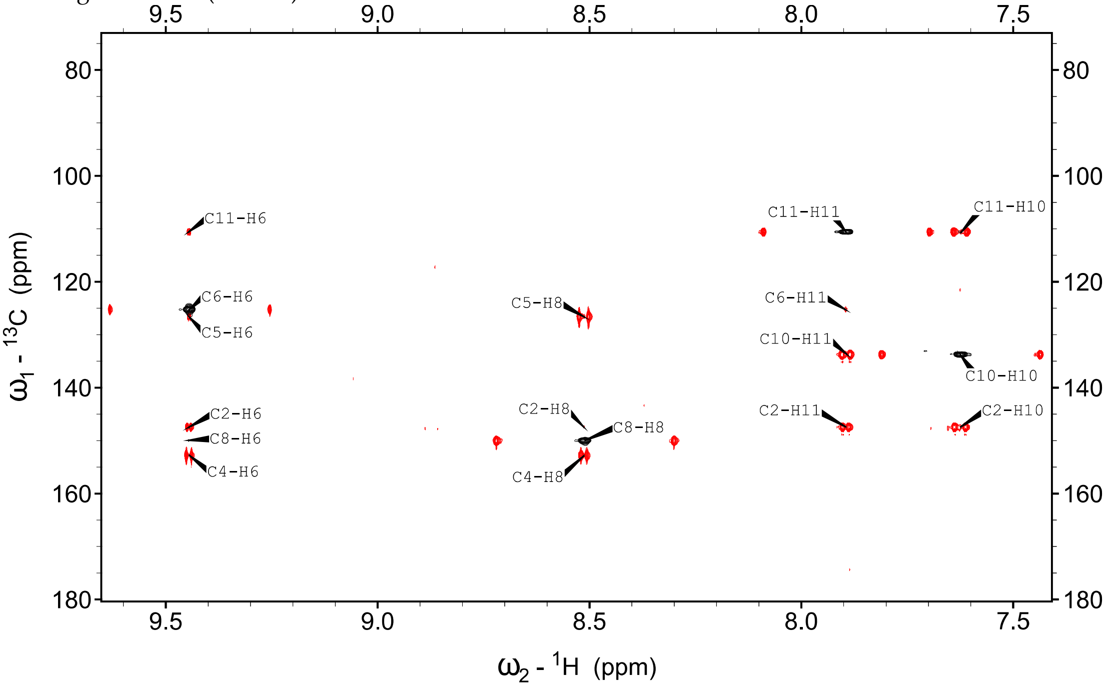

Figure 4S. Contour plots of ^{1}H, ^{13}C correlation spectra of 1,N^2-etheno-2-aminopurine (**1**) in DMSO-d$_6$ (at ^{1}H frequency of 500 MHz, at 25 °C). Contours are plotted: for HSQC (black) and for 2 Hz optimized HSQMBC (red). All peaks are marked at the center of the multiplet and labeled according to the assigned nuclei (Table 2).

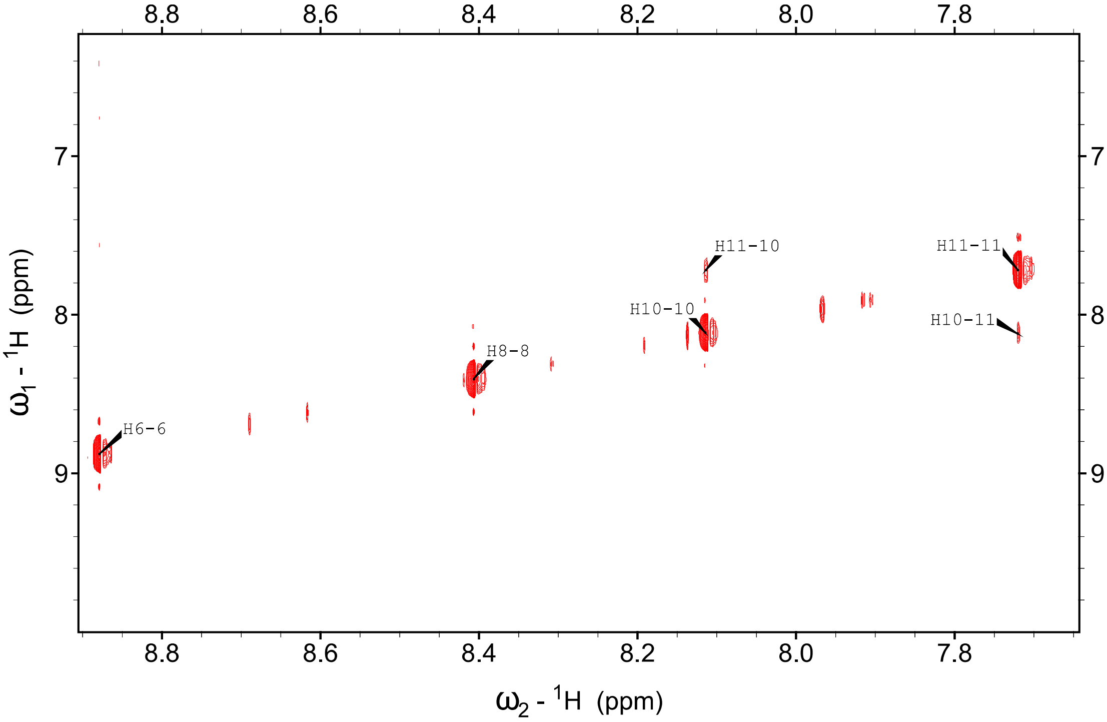

Figure 5S. Contour plot of the ^{1}H, ^{1}H COSY spectrum of N^2,3-etheno-2-aminopurine (**2**) in DMSO-d$_6$ (at ^{1}H frequency of 500 MHz, at 25 °C). All peaks are marked at the center of the multiplet and labeled according to the assigned nuclei (Table 2).

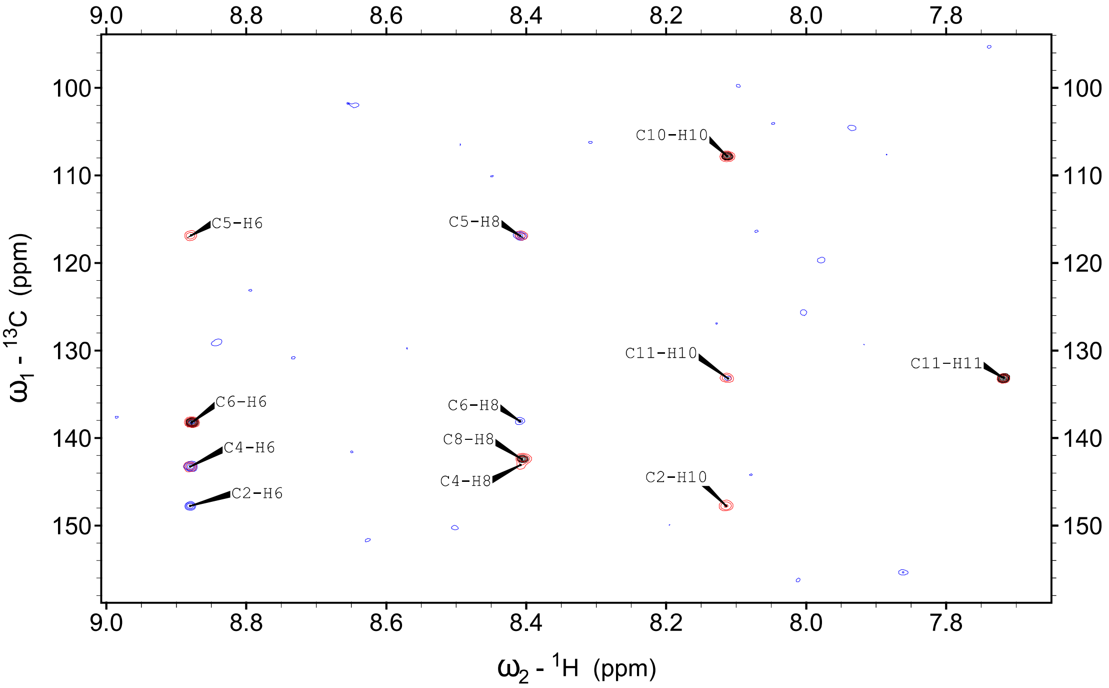

Figure 6S. Contour plots of ^{1}H, ^{13}C correlation spectra of N[2],3-etheno-2-aminopurine (**2**) in DMSO-d6 (at ^{1}H frequency of 500 MHz, at 25 °C). Contours are plotted: for HSQC (black), for 8 Hz optimized decoupled HSQMBC (red), for 2 Hz optimized decoupled HSQMBC (blue). All peaks are marked at the center of the multiplet and labeled according to the assigned nuclei (Table 2).

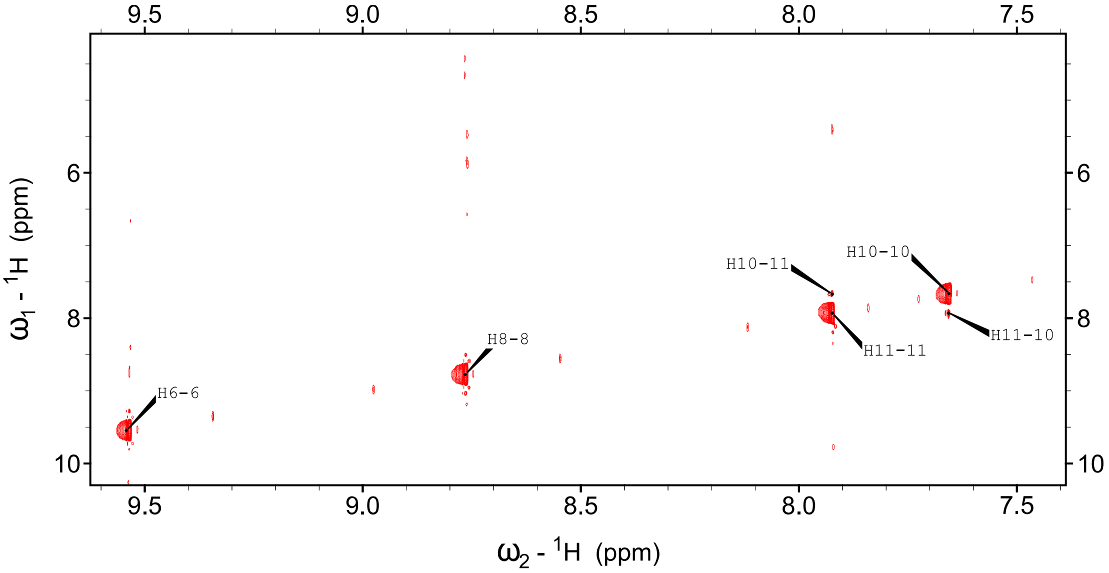

Figure 7S. Aromatic region of the contour plot of the ^{1}H, ^{1}H COSY spectrum of 1,N[2]-etheno-2-aminopurine-N9-riboside (**3**) in DMSO-d6 (at ^{1}H frequency of 500 MHz, at 25 °C). All peaks are marked at the center of the multiplet and labeled according to the assigned nuclei (Table 2).

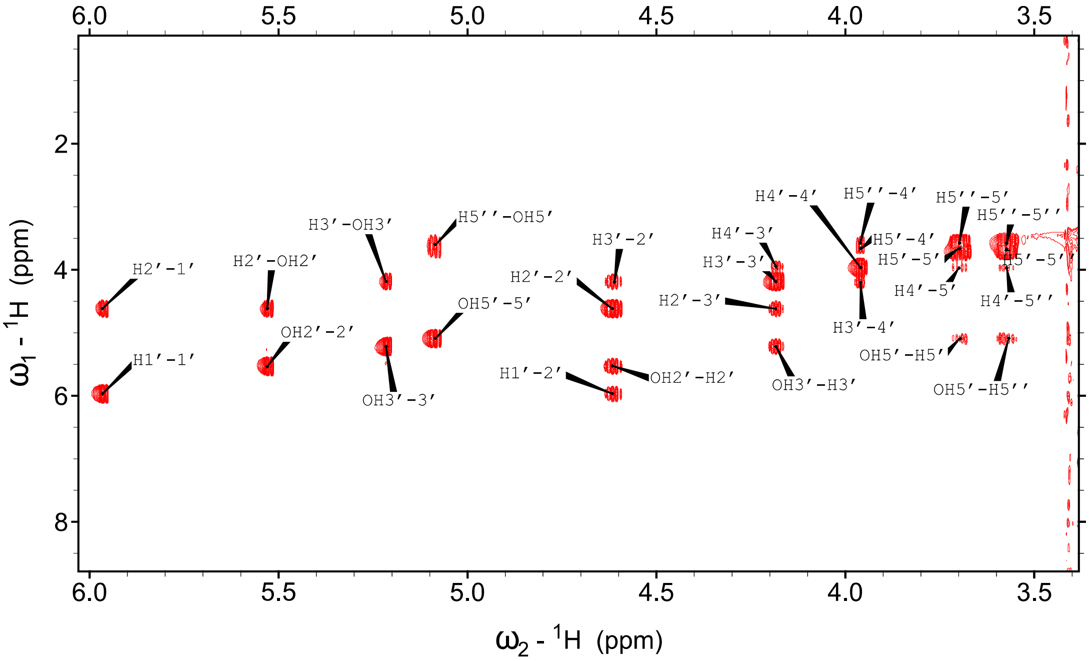

Figure 8S. Aliphatic region of the contour plot of the ^{1}H, ^{1}H COSY spectrum of 1,N^2-etheno-2-aminopurine-9-riboside (**3**) in DMSO-d$_6$ (at ^{1}H frequency of 500 MHz, at 25 °C). All peaks are marked at the center of the multiplet and labeled according to the assigned nuclei (Table 2).

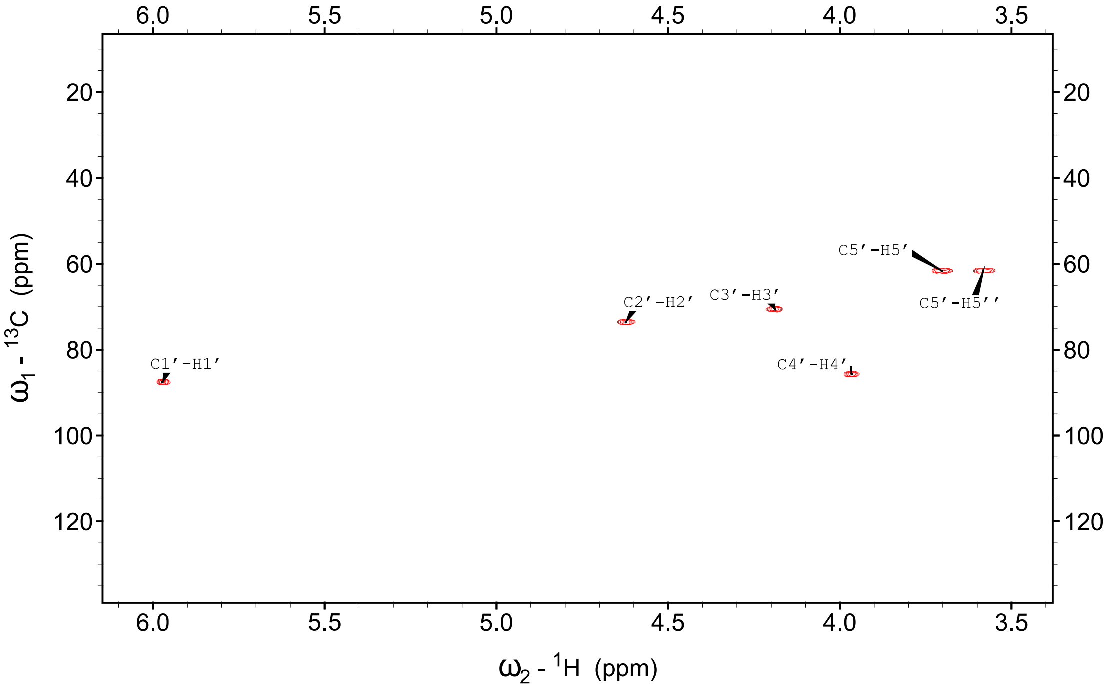

Figure 9S. Aliphatic region of the contour plot of ^{1}H, ^{13}C HSQC spectrum of 1,N^2-etheno-2-aminopurine-N9-riboside (**3**) in DMSO-d6 (at ^{1}H frequency of 500 MHz, at 25 °C). All peaks are marked at the center of the multiplet and labeled according to the assigned nuclei (Table 2).

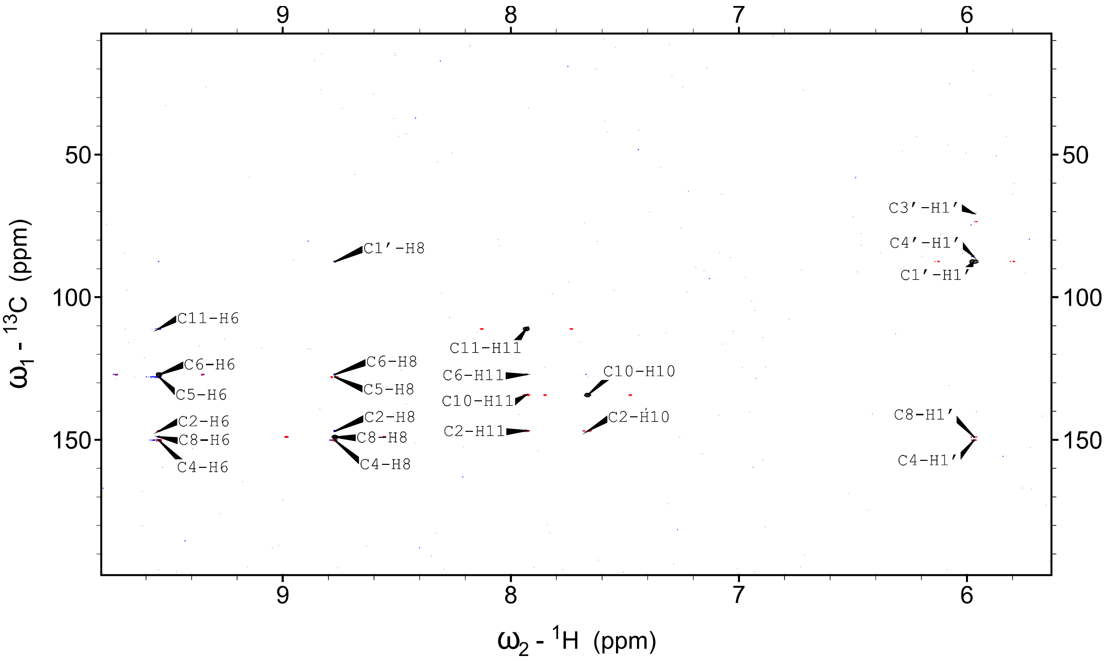

Figure 10S. Aromatic and H(1′)/C(1′) region of the contour plots of ^{1}H, ^{13}C correlation spectra of 1,N^2-etheno-2-aminopurine-N9-riboside (**3**) in DMSO-d$_6$ (at ^{1}H frequency of 500 MHz, at 25 °C). Contours are plotted: for HSQC (black), for 8 Hz optimized HSQMBC (red), for 2 Hz optimized HSQMBC (blue). All peaks are marked at the center of the multiplet and labeled according to the assigned nuclei (Table 2).

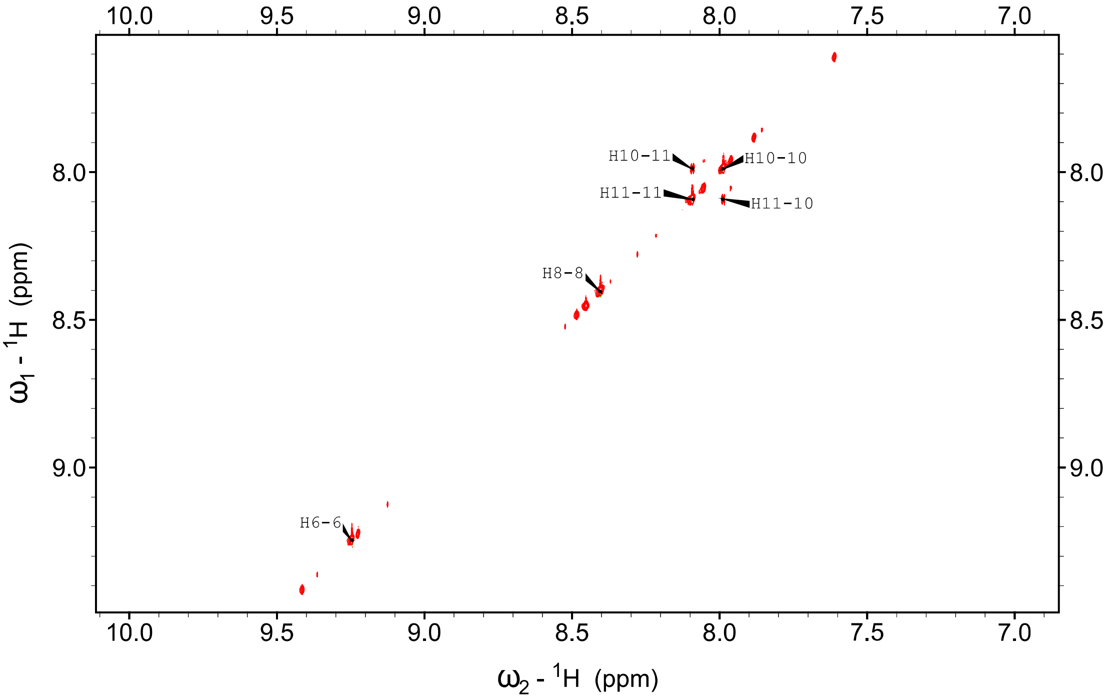

Figure 11S. Aromatic region of the contour plot of the ^{1}H, ^{1}H COSY spectrum of 1,N^2-etheno-2-aminopurine-N^2-riboside (**4**) in DMSO-d$_6$ (at ^{1}H frequency of 500 MHz, at 25 °C). All peaks are marked at the center of the multiplet and labeled according to the assigned nuclei (Table 2).

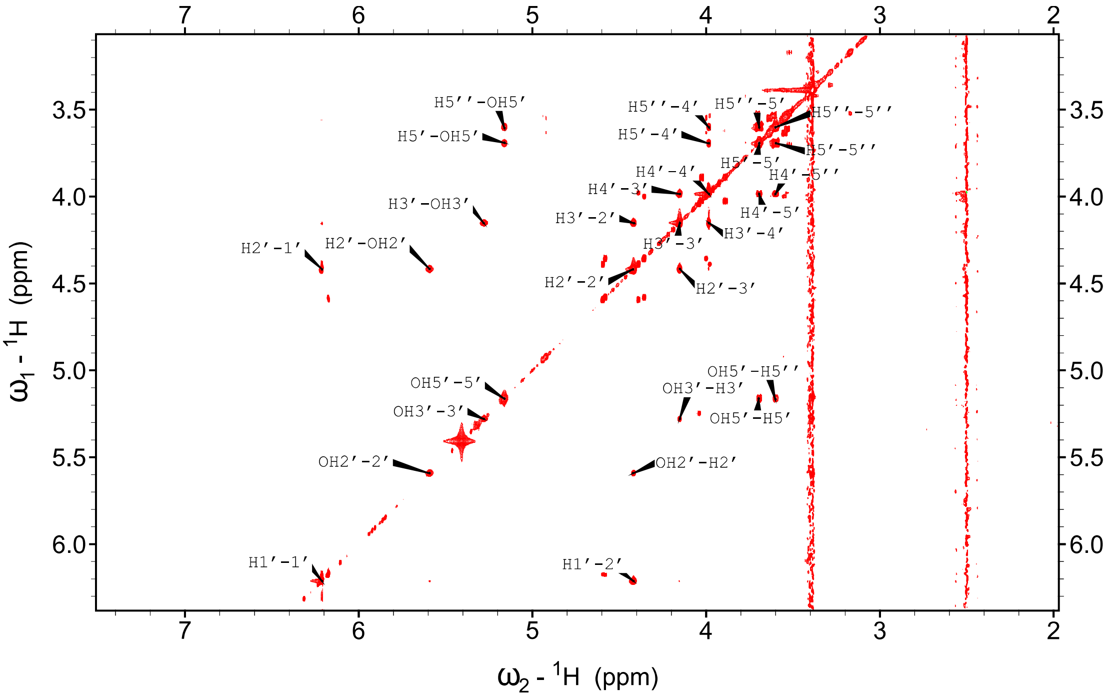

Figure 12S. Aliphatic region of the contour plot of the ^{1}H, ^{1}H COSY spectrum of 1,N^2-etheno-2-aminopurine-N^2-riboside (**4**) in DMSO-d$_6$ (at ^{1}H frequency of 500 MHz, at 25 °C). All peaks are marked at the center of the multiplet and labeled according to the assigned nuclei (Table 2).

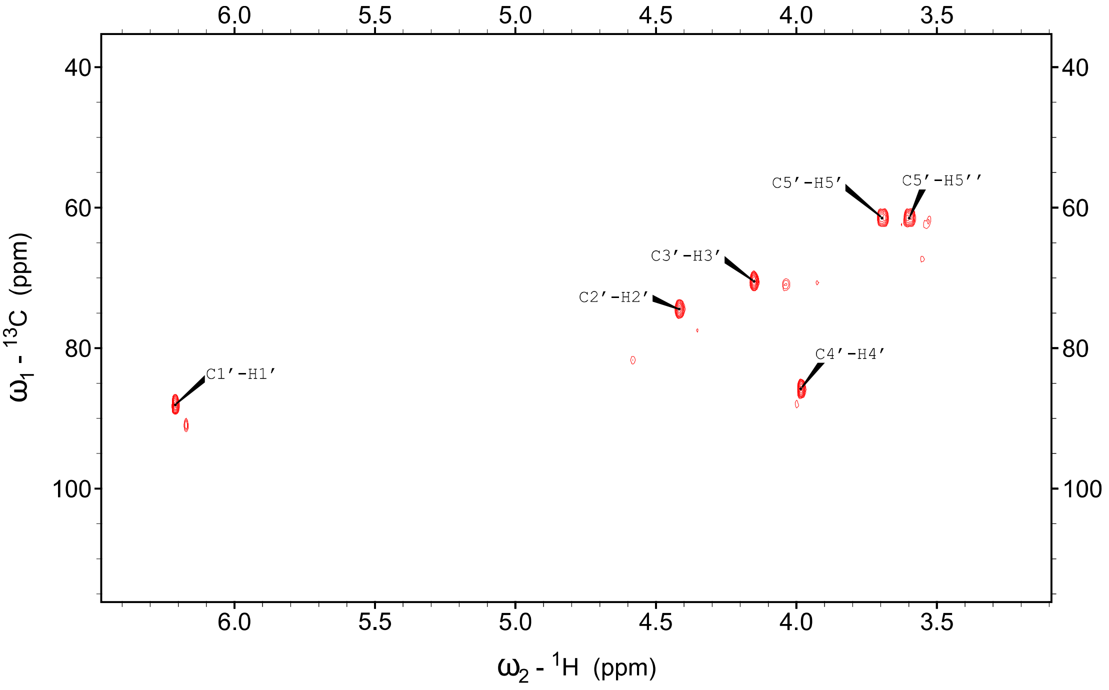

Figure 13S. Aliphatic region of the contour plot of ^{1}H, ^{13}C HSQC spectrum of 1,N^2-etheno-2-aminopurine-N^2-riboside (**4**) in DMSO-d6 (at ^{1}H frequency of 500 MHz, at 25 °C). All peaks are marked at the center of the multiplet and labeled according to the assigned nuclei (Table 2).

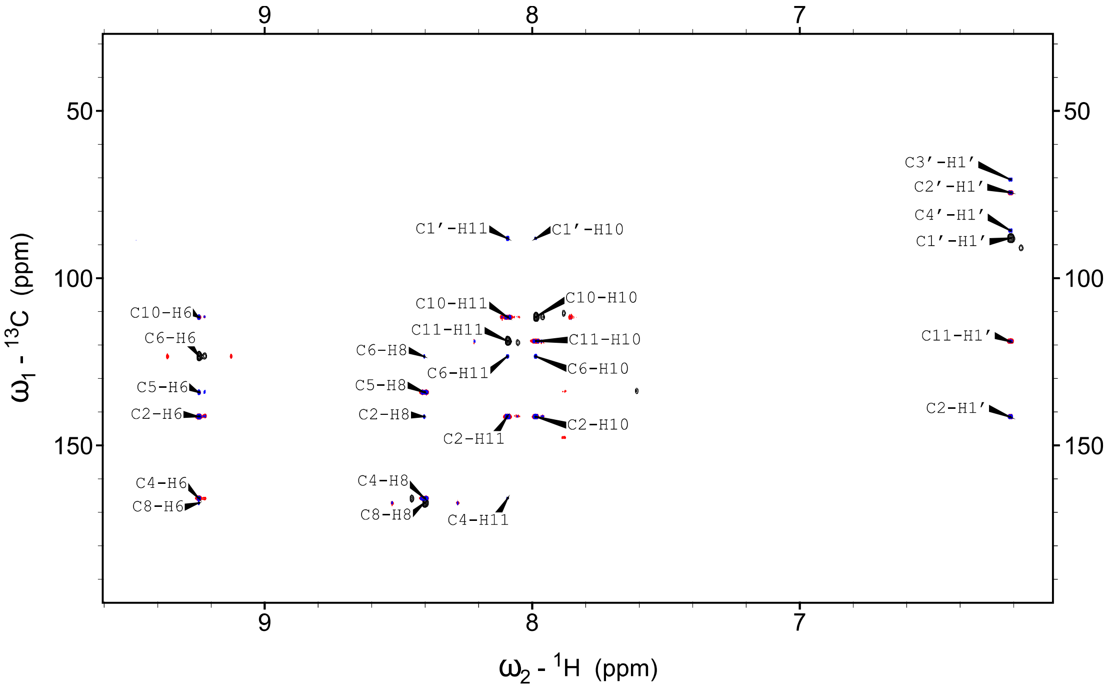

Figure 14S. Aromatic and H(1′)/C(1′) region of the contour plots of ^{1}H, ^{13}C correlation spectra of 1,N^2-etheno-2-aminopurine-N^2-riboside (**4**) in DMSO-d$_6$ (at ^{1}H frequency of 500 MHz, at 25 °C). Contours are plotted: for HSQC (black), for 8 Hz optimized HMBC (red), for 2 Hz optimized HSQMBC (blue). All peaks are marked at the center of the multiplet and labeled according to the assigned nuclei (Table 2).

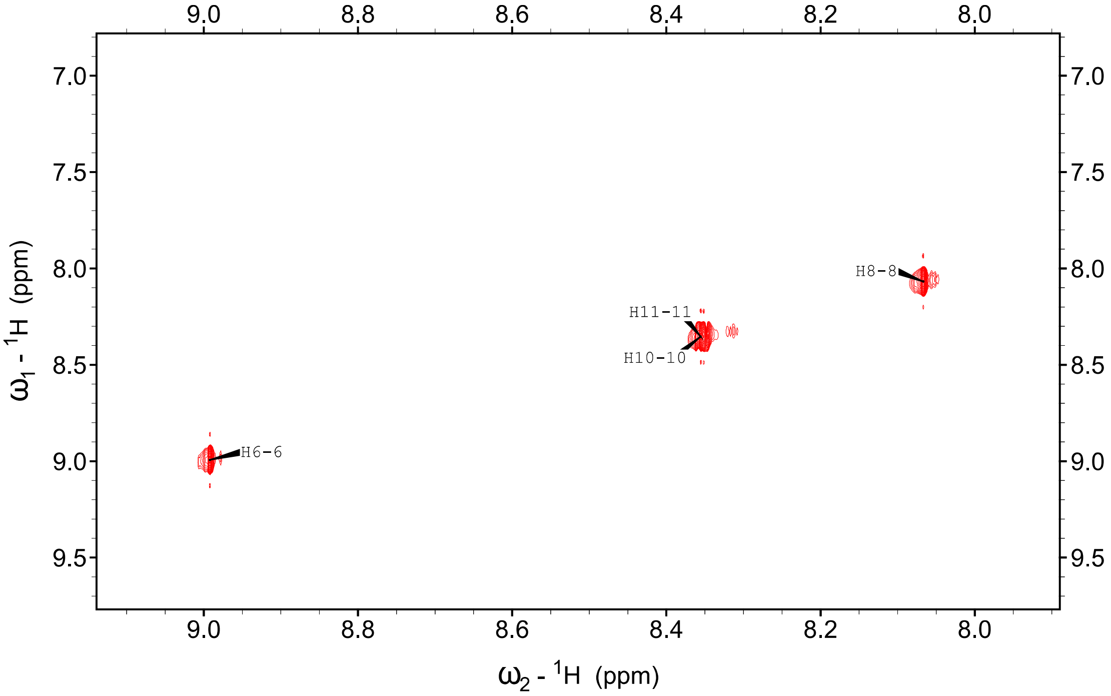

Figure 15S. Aromatic region of the contour plot of the ^{1}H, ^{1}H COSY spectrum of N^2,3-etheno-2-aminopurine-N^2-riboside (**5**) in DMSO-d$_6$ (at ^{1}H frequency of 500 MHz, at 25 °C). All peaks are marked at the center of the multiplet and labeled according to the assigned nuclei (Table 2).

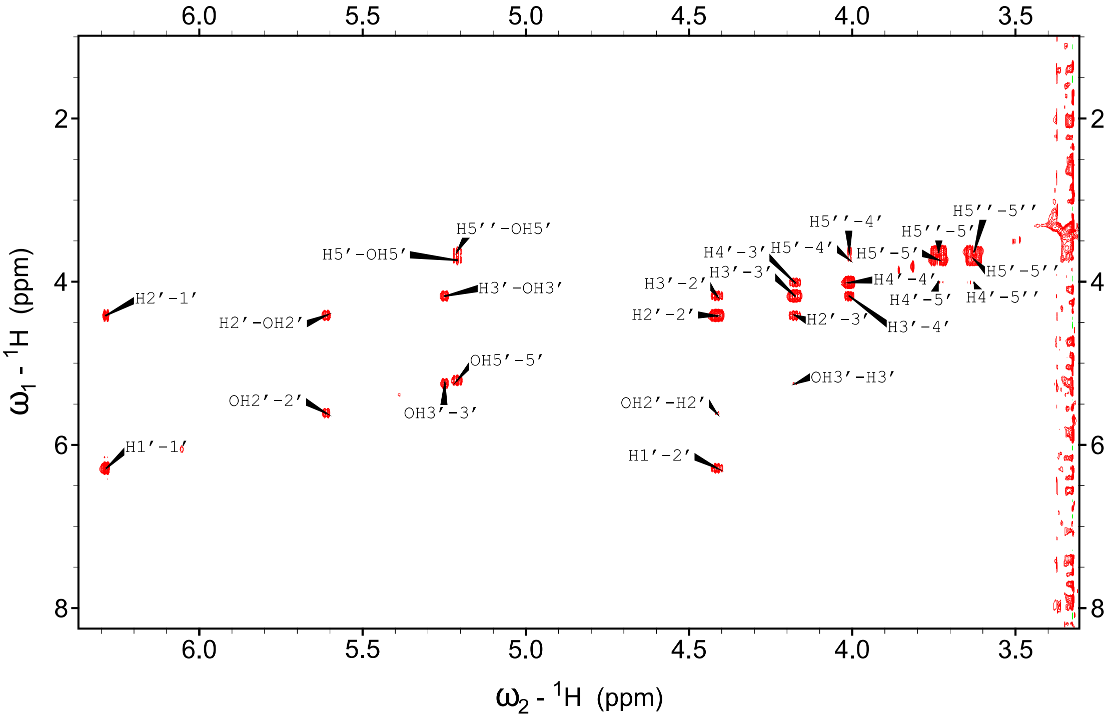

Figure 16S. Aliphatic region of the contour plot of the ^{1}H, ^{1}H COSY spectrum of N^2,3-etheno-2-aminopurine-N^2-riboside (**5**) in DMSO-d$_6$ (at ^{1}H frequency of 500 MHz, at 25 °C). All peaks are marked at the center of the multiplet and labeled according to the assigned nuclei (Table 2).

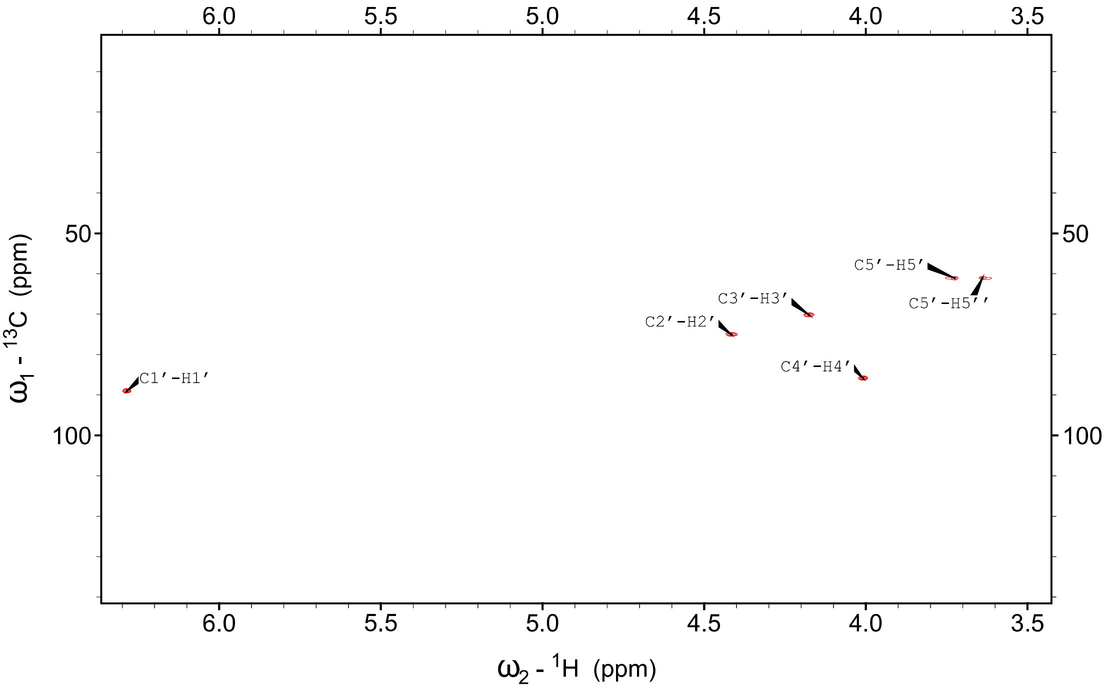

Figure 17S. Aliphatic region of the contour plot of ^{1}H, ^{13}C HSQC spectrum of N^2,3-etheno-2-aminopurine-N^2-riboside (**5**) in DMSO-d6 (at ^{1}H frequency of 500 MHz, at 25 °C). All peaks are marked at the center of the multiplet and labeled according to the assigned nuclei (Table 2).

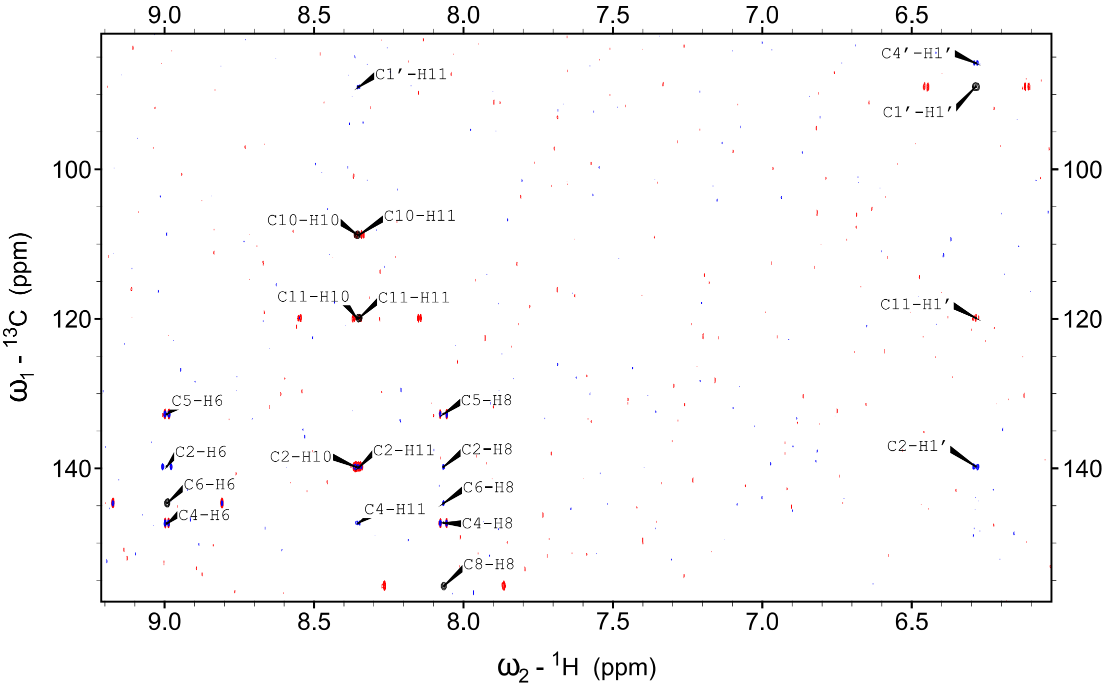

Figure 18S. Aromatic and H(1′)/C(1′) region of the contour plots of ^{1}H, ^{13}C correlation spectra of N^{2},3-etheno-2-aminopurine-N^{2}-riboside (**5**) in DMSO-d_6 (at ^{1}H frequency of 500 MHz, at 25 °C). Contours are plotted: for HSQC (black), for 8 Hz optimized HSQMBC (red), for 2 Hz optimized HSQMBC (blue). All peaks are marked at the center of the multiplet and labeled according to the assigned nuclei (Table 2).

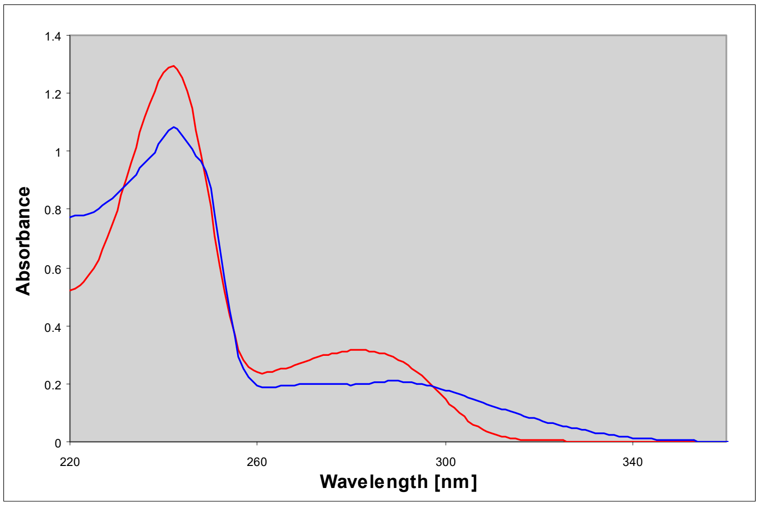

Figure 19S. UV absorption of 1,N^{6}-etheno-tubercidine in neutral (blue line) and acidic (red) aqueous medium.

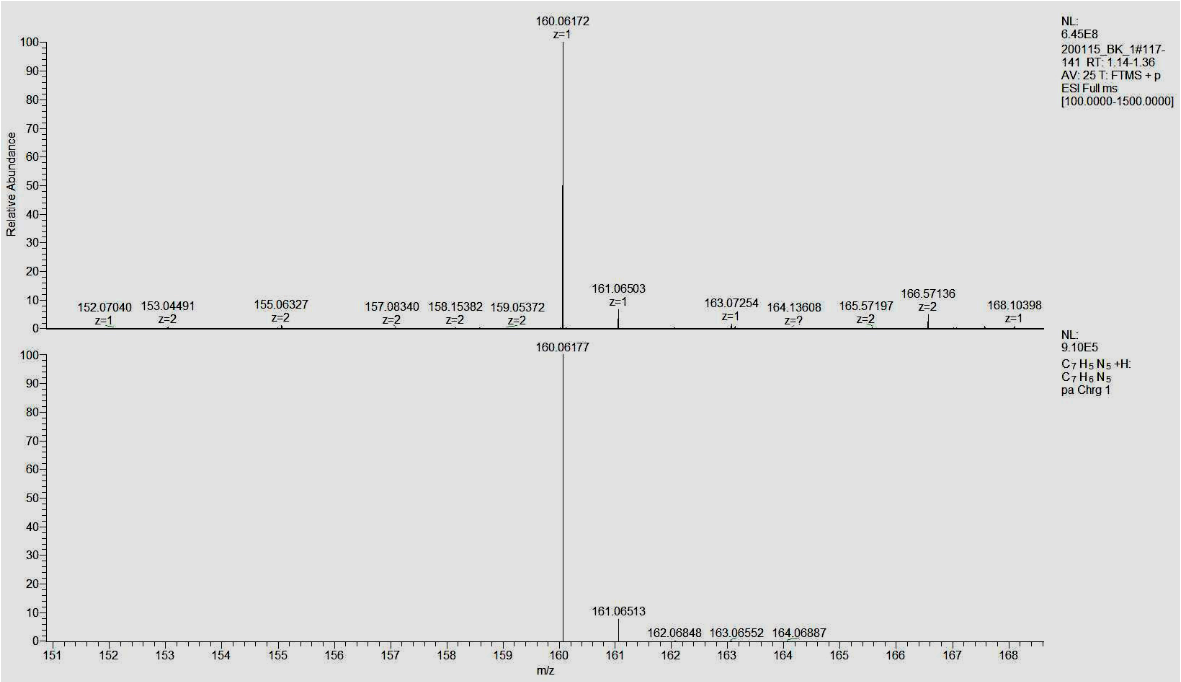
NL: 6.45E8
200115_BK_1#117-141 RT: 1.14-1.36 AV: 25 T: FTMS + p ESI Full ms [100.0000-1500.0000]
NL: 9.10E5
C7 H5 N5 +H: C7 H6 N5 pa Chrg 1
Relative Abundance
m/z
160.06172 z=1
160.06177

(a)

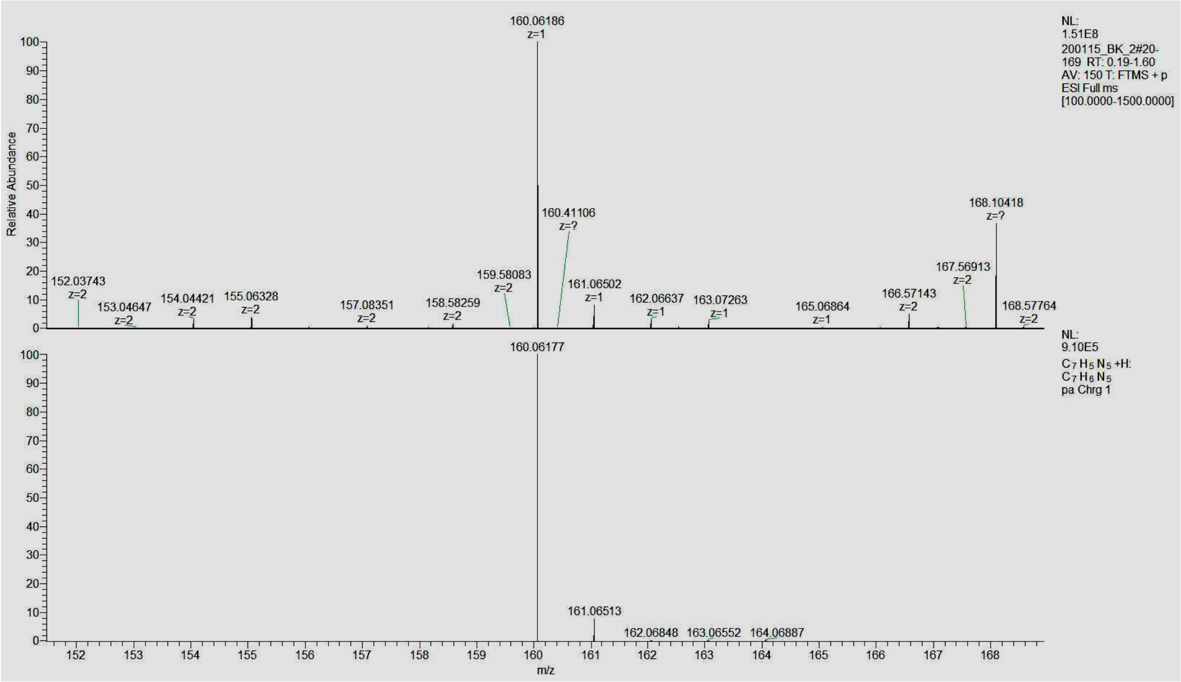
NL: 1.51E8
200115_BK_2#20-169 RT: 0.19-1.60 AV: 150 T: FTMS + p ESI Full ms [100.0000-1500.0000]
NL: 9.10E5
C7 H5 N5 +H: C7 H6 N5 pa Chrg 1
Relative Abundance
m/z
160.06186 z=1
160.06177

(b)

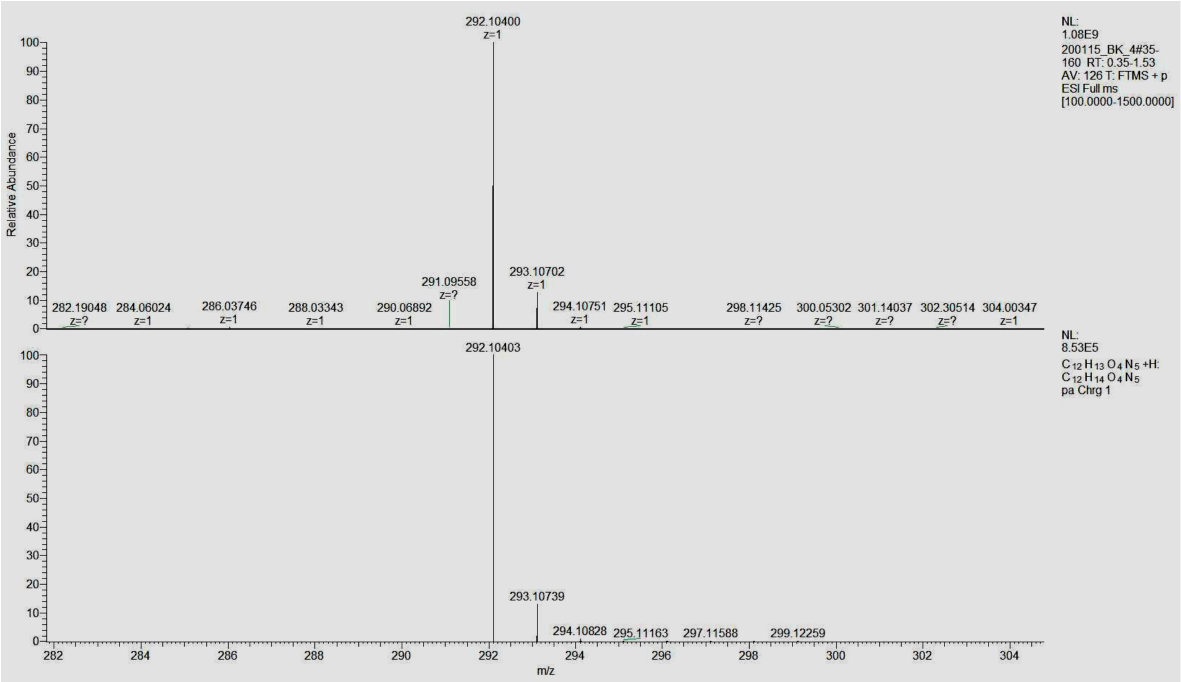

(c)

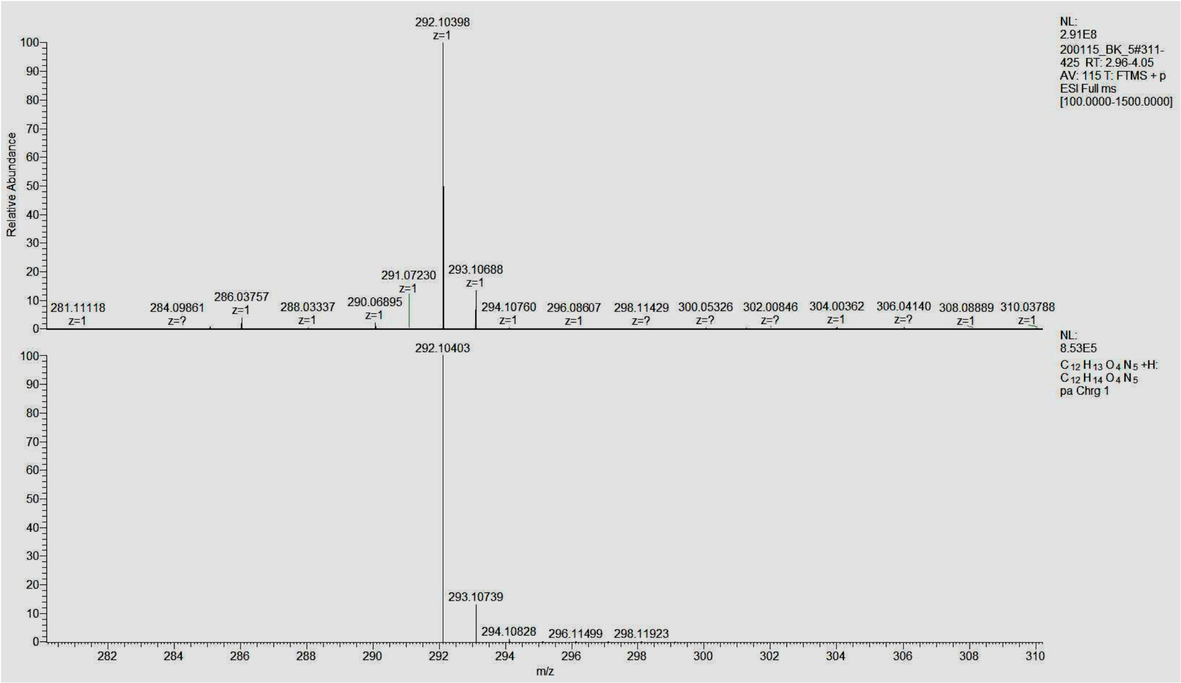

(d)

Figure 20S. Mass Spectroscopy (m/z determination) of the new compounds described in this paper: (a) compound **1**, (b) compound **2**, (c) compound **4** (d) compound **4.** Calculated m/z values are shown in lower panels.

Below is a quotation from reference [24]: Bhat et al., *J. Carbohydrates, Nucleosides and Nucleotides* 7 (**1980**), 333-345).

spectrophotometer using TMS as an internal standard. The pmr spectra of compounds 13 and 14 were recorded on a EM-390 MHz spectrophotometer using TMS as the internal standard. Chemical shifts are expressed as δ (parts per million) relative to the internal standard. Thin layer chromatography was run on glass plates coated (0.25 mm) with SilicAR 7 GF (Mallinckrodt). The infrared spectra were recorded on a Beckman IR-8 spectrometer in pressed potassium bromide disks.

7-(β-D-ribofuranosyl)imidazo[1,2-c]pyrrolo[3,2-e]pyrimidine (8).

Tubercidin (1, 2.0 g, 7.51 mmole) and freshly distilled chloroacetaldehyde (30 ml) were mixed and stirred at 30° for 18 hr. The solvent was removed *in vacuo* and the resulting residue was co-evaporated with ethanol (3 x 20 ml). The crude solid was dissolved in hot ethanol (40 ml), filtered and the solution allowed to stand at 5° for 18 hr. The white crystals which separated were collected by filtration and dried at 125° *in vacuo* for 6 hr, to afford 2.3 g of 8 (93%), mp 202-204° (dec.), pmr (DMSO-d_6): δ 9.72 (s, 1, H_5), 8.63 and 8.32 (two sets of doublets, 2, $J_{2,3}$ = 2 Hz, H_2 and H_3), 8.2 and 7.36 (two sets of doublets, 2, $J_{8,9}$ = 3 Hz, H_8 and H_9), 6.47 (d, 1, $J_{1',2'}$ = 5 Hz, $H_{1'}$). Anal. Calcd for $C_{13}H_{14}N_4O_4 \cdot HCl$: C, 47.71; H, 4.59; N, 17.13. Found: C, 47.61; H, 4.81; N, 17.46.

9-Cyano-7-(β-D-ribofuranosyl)imidazo[1,2-c]pyrrolo[3,2-e]pyrimidine (9).

A mixture of toyocamycin (2, 2.0 g, 6.87 mmole) and freshly distilled chloroacetaldehyde (30 ml) was stirred at 30° for 20 hr. The solvent was evaporated to dryness *in vacuo* and the residue co-evaporated with ethanol (3 x 30 ml). This crude solid was triturated
